# Maternal and fetal outcomes in subsequent pregnancies after peripartum cardiomyopathy: A systematic review and meta‐analysis

**DOI:** 10.1111/aogs.15117

**Published:** 2025-04-30

**Authors:** Rebecca Man, Jack Le Vance, Hafsa Abdullahi, Clare Davenport, Mengshi Yuan, Laura Ormesher, Sana Usman, James Castleman, Caroline Fox, Lucy Hudsmith, R. Katie Morris, Victoria Hodgetts Morton

**Affiliations:** ^1^ Department of Applied Health Sciences, School of Health Sciences, College of Medicine and Health University of Birmingham Birmingham UK; ^2^ Birmingham Women's Hospital Birmingham UK; ^3^ Epsom and St Helier University Hospitals London UK; ^4^ Queen Elizabeth Hospital Birmingham UK; ^5^ Maternal & Fetal Health Research Centre, Division of Developmental Biology and Medicine University of Manchester Manchester UK; ^6^ Saint Mary's Hospital Manchester University NHS Foundation Trust Manchester UK; ^7^ Queen Charlotte's and Chelsea Hospital London UK; ^8^ Department of Metabolism, Digestion and Reproduction, Institute of Reproductive Developmental Biology Imperial College London London UK

**Keywords:** fetal growth restriction, heart disease, maternal mortality, obstetric medicine, PPCM, pre‐eclampsia, preterm birth

## Abstract

**Introduction:**

We aim to determine the pregnancy and cardiovascular outcomes of subsequent pregnancies following a previous diagnosis of peripartum cardiomyopathy.

**Material and Methods:**

Medline, Embase, CINAHL, and Web of Science were searched from inception to November 2023. Primary research studies of any design providing data for any of our outcomes of interest with greater than five subsequent pregnancies were eligible for inclusion. Primary outcomes included relapse of cardiac failure in the first subsequent pregnancy and death during any subsequent pregnancy. Secondary outcomes included a range of maternal and fetal outcomes. Proportional meta‐analysis, applying a random effects model, was performed using R software.

**Results:**

Twenty‐nine studies involving 779 women were included, performed across 13 countries. The risk of relapse of cardiac failure in the first subsequent pregnancy (20 studies, 376 women) was 32%, 95% confidence interval [CI] 0.23–0.43. In those with recovered (11 studies, 123 pregnancies) and non‐recovered (10 studies, 55 pregnancies) cardiac function at subsequent pregnancy outset, the risk of cardiac failure relapse was 24%, 95% CI 0.16–0.35 and 36%, 95% CI 0.24–0.50, respectively. There was a high chance of preterm birth <37 weeks (12 studies, 212 pregnancies) at 22%, 95% CI 0.17–0.29.

**Conclusions:**

Subsequent pregnancy after peripartum cardiomyopathy presents significant maternal and fetal risks. This study provides quantification of risks to begin to fill the current evidence gap, however is limited by the paucity of existing primary research investigating this population. Robust observational studies of current practice are needed to provide answers in specific populations.

## Graphical abstract

Our systematic review and meta‐analysis found that subsequent pregnancy after peripartum cardiomyopathy presents significant maternal and fetal risks. This study provides quantification of risks to begin to fill the current evidence gap, however is limited by a paucity of existing data.

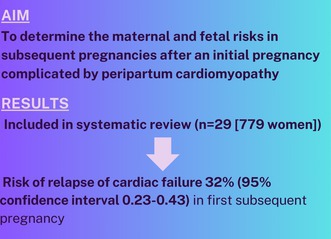

**Key message**

There is a lack of evidence exploring subsequent pregnancy outcomes after a previous pregnancy complicated by peripartum cardiomyopathy. In subsequent pregnancy, our meta‐analysis demonstrates a 32% risk of cardiac failure relapse.

## 1 INTRODUCTION

Peripartum cardiomyopathy is a rare complication of pregnancy that can be life‐threatening. It is a diagnosis of exclusion and is defined as cardiomyopathy which presents with left ventricular systolic dysfunction towards the end of pregnancy or postpartum, where no alternative cause is established.^1^ The left ventricle may or may not be dilated, however the ejection fraction is nearly always under 45%.^1^ Estimates on the prevalence of peripartum cardiomyopathy vary significantly across studies and populations, with incidence in the United States and Scotland estimated at one in 968 and one in 4950 births, respectively.^2^, ^3^ Well‐established risk factors include pre‐existing or pregnancy‐related hypertensive disorders, African ethnicity, obesity, and diabetes.^3^, ^4^ Genetic factors are also implicated, with an increased awareness of these in recent years and a notable overlap in variants associated with other types of cardiomyopathy.^5^, ^6^

Women who have experienced peripartum cardiomyopathy in an index pregnancy often express concerns about the safety of a subsequent pregnancy, both in terms of maternal and fetal risks. While there are existing cohorts documenting the outcomes of subsequent pregnancies, these are few and mostly include small numbers of women. In the absence of robust data, consultations will often rely on specialist expertise and experience, or very limited evidence. It is imperative that women are provided with the best possible information in order to make informed decisions about future pregnancies. Consideration must also be given on how best to individualize these risks; to take into account the woman's health prior to, or at the beginning of the subsequent pregnancy. Recovery from peripartum cardiomyopathy after the index pregnancy is highly variable, with some women demonstrating a full recovery of their ejection fraction, others maintaining a stable but reduced ejection fraction and a proportion of women progressing to be listed for cardiac transplant or dying from cardiac complications.^7^ The impact of cardiac recovery status on outcomes in subsequent pregnancies is therefore imperative to consider.

One existing systematic review on this subject provides a mostly narrative synthesis on a subset of published studies, however notably pregnancy outcomes are lacking and there is a paucity of appropriate meta‐analysis.^8^ We therefore aim to provide a comprehensive systematic review, including all available studies with a wide range of both maternal and fetal outcomes.

The objective of this systematic review and meta‐analysis is to determine the risk of adverse maternal, fetal, and cardiovascular outcomes in subsequent pregnancies after an index pregnancy complicated by peripartum cardiomyopathy.

## 2 MATERIAL AND METHODS

### 2.1 Registration

This systematic review and meta‐analysis were prospectively registered on PROSPERO: CRD42023481440.

### 2.2 Eligibility criteria and outcomes

Primary research studies of any design providing data for any of our specified outcomes with greater than five subsequent pregnancies were eligible for inclusion. To determine a prior diagnosis of peripartum cardiomyopathy, studies were included where the authors stated the diagnosis as peripartum cardiomyopathy and/or where congestive cardiac failure occurred in the latter stages of pregnancy or postpartum, without any pre‐existing cardiac disease and without cardiac failure found to be of any other cause. Where a cohort that was likely the same or overlapping appeared in more than one study, only the most complete dataset was used for each outcome to avoid potential duplication. Studies published at any time point, undertaken in any country and in any language were eligible for inclusion. Studies were excluded if there were five or fewer subsequent pregnancies after removal of pregnancies clearly resulting in miscarriage or termination. Studies were excluded if they only included women who had a relapse of peripartum cardiomyopathy in a subsequent pregnancy (and those who did not experience relapse were excluded from outset). Where the study was only published in abstract format and was not available as a full text then the study was excluded.

Primary outcomes were relapse of cardiac failure in the first subsequent pregnancy and death during any subsequent pregnancy (limited to the time during a subsequent pregnancy and not after birth). Secondary outcomes included relapse of cardiac failure in the first subsequent pregnancy in those with recovered and non‐recovered cardiac function at outset, death overall and by recovered and non‐recovered cardiac function at outset, need for cardiac transplant, postpartum hemorrhage, pregnancy‐induced hypertension, pre‐eclampsia, hypertensive disorder composite (to include pregnancy‐induced hypertension and pre‐eclampsia), preterm birth <37 weeks, preterm birth <34 weeks, preterm birth <28 weeks, stillbirth, neonatal death, fetal growth restriction or growth <3rd centile, small for gestational age or growth <10th centile, birthweight and gestational age at birth. Outcomes and determination of recovery versus non‐recovery of cardiac function prior to pregnancy were as defined by the authors of the primary studies. Definitions used by study authors of peripartum cardiomyopathy and of recovered versus non‐recovered cardiac status were recorded in the study characteristics table (Table 1). Subgroup analyses for primary outcomes were performed by countries' World Bank income level which categorizes countries as low income, lower‐middle income, upper‐middle income, or high income.^9^ Where a study was conducted in more than one country, the World Bank income level used for subgroup analysis was determined by the country with the lowest classification. Where possible, the pregnancies resulting in miscarriage or termination were removed from outcome data.

**TABLE 1:** Study characteristics (included studies).

Author and year (Ref)	Years included in study	Country	Study design	Number of women (*n*=) in study with a subsequent pregnancy after PPCM	Aim of study	SP as focus or secondary?	Definition of PPCM	Definition of recovered vs non‐recovered cardiac function	Definition of relapse in SP used
Albanesi Filho 1999^10^	1976–1996	Brazil	Prospective cohort	*N* = 12	To assess the effect of SP after PPCM on maternal and fetal outcome	SP as focus	Heart failure of unknown cause occurring in the latter 3 months of pregnancy or first 6 months post‐delivery, without underlying cardiovascular disease	Left ventricular dysfunction: signs and symptoms of heart failure and increased heart size in one or more exams	Decline in NYHA FC
Avila 2002^11^	1990–1998	Brazil	Prospective cohort	*N* = 18	To evaluate the course of SP in women with previous PPCM, to evaluate risk factors, and to compare with outcomes of women with idiopathic dilated cardiomyopathy	SP as focus	PPCM criteria as per WHO committee 1980, modified in 1996	Left ventricular dysfunction EF <55% although not explicit in methods	Congestive heart failure (not further defined)
Chapa 2005^12^	1998–2001	USA	Retrospective cohort	*N* = 6	To investigate whether echocardiography findings at diagnosis of PPCM predict future cardiac dysfunction	SP as secondary	No other cause of heart failure found in late gestation. Fractional shortening <30% and left ventricular end diastolic dimension ≥4.8 cm at diagnosis	Recovered: Fractional shortening >30% or LV end diastolic dimension <4.8 cm	Recurrence of heart failure symptoms or decline in LV function
Codsi 2018^13^	2000–2017	USA	Retrospective cohort	*N* = 25	To describe outcomes in SPs of patients with PPCM and to report index pregnancy characteristics	SP as focus	(1) New cardiac failure in the final month of pregnancy or within 5 months postpartum, (2) no other cause found for cardiac failure, (3) absence of structural cardiac disease before final month of pregnancy, (4) LV systolic dysfunction with LVEF<45%	Recovered LVEF ≥50%, non‐recovered LVEF <50%	(1) decrease of LVEF to ≤45% or (2) absolute decrease in LVEF ≥10% in patients with LVEF ≤50% or less at SP outset
de Souza 2001^14^	1990–1999	Brazil	Prospective cohort	*N* = 7	To evaluate LV systolic function after a new pregnancy in women with PPCM	SP as focus	Clinical symptoms and signs of cardiac failure in the last trimester of pregnancy or up to 6 months postpartum in previously healthy women. Excluded those with congenital heart disease and valvular disease or any illness that could impact cardiac function/structure		Deterioration of left ventricular function
Douglass 2021^15^	1970–2014	USA	Case‐control	*N* = 23	To determine PPCM incidence and outcomes of PPCM and to compare to a control group	SP as secondary	(1) New heart failure in the last month of pregnancy or within 5 months postpartum, (2) no other cause for cardiac failure (3) no recognizable cardiac disease prior to the final month of pregnancy, (4) LV systolic dysfunction with LVEF ≤45% on echocardiogram	Recovered LVEF ≥50%, non‐recovered LVEF <50%	
Elkayam 2001^16^	Questionnaire sent 1997–1998	USA, South Africa	Retrospective cohort (obtained via sending a questionnaire to physicians)	*N* = 44	Assess outcomes of SP in women with PPCM	SP as focus	New congestive cardiac failure during the last 6 months of pregnancy or within 6 months postpartum, the absence of any other cause of cardiac failure and reduced LV function (LVEF <40%)	Recovered LVEF ≥50%, non‐recovered LVEF <50%	>20% decrease in LVEF
Fett 2010^17^	2003–2009	USA, Haiti	Some prospective cohort, some via self‐identification via internet support group and then retrospective cohort	*N* = 56	To determine the risk of heart failure relapse in a SP in women with previous PPCM. To determine whether certain factors lower the risk.	SP as focus	(1) initial development of cardiac failure during the final month of pregnancy or within 5 months post‐delivery; (2) no other cause of cardiac failure; (3) no previous cardiac diagnosis; and (4) systolic heart failure with LVEF ≤45%	Recovered LVEF ≥55%, non‐recovered LVEF <55%	Reduction in LVEF to ≤45% and/or deterioration in NYHA FC. Decline in LVEF ≥10% where LVEF ≤45% at baseline.
Fett 2003^18^	2000–2002	Haiti	Case‐control	*N* = 8	To identify risk factors for PPCM	SP as secondary	Definition of PPCM not clear from methods		Recurrent congestive heart failure
Ford 1998^19^	1969–1972 index cases, followed up 1993–1995	Nigeria	Retrospective cohort	*N* = 171	To follow‐up survivors after an index PPCM pregnancy	SP as secondary	Cardiac failure with symptoms starting during pregnancy or up to 6 months after birth, no previous history of heart failure other than the peripartum cardiac failure and no underlying cause found. Clinical/radiological evidence of systematic venous/pulmonary congestion.		
Goland 2022^20^	2007–2019	Israel	Prospective cohort	*N* = 50	To describe the impact of SP on LV function and outcomes in women with PPCM	SP as focus	Idiopathic cardiomyopathy presenting during pregnancy or within 5 months postpartum. Echocardiographic LVEF 45% (presumed below 45%)	Recovered LVEF ≥55%, non‐recovered LVEF <55%	Decrease of LVEF to ≤45% or decrease in LVEF of ≥10% in women with persistent LV dysfunction before SP
Guldbrandt Hauge 2017^21^	2005–2016	Denmark	Retrospective cohort	*N* = 13	To describe SP outcome for a Danish cohort of women with PPCM	SP as focus	European Society of Cardiology criteria used	Recovered LVEF ≥55%, non‐recovered LVEF <55%	Decrease of LVEF to ≤45%
Habli 2008^22^	2000–2006	USA	Retrospective cohort	*N* = 37	To assess the prognostic value of EF at index and SP	SP as secondary	Congestive heart failure during final month of pregnancy/first 6 months postpartum, no other cause for cardiac failure and reduced LV function (LVEF <40%)/fractional shortening <30% with an end‐diastolic dimension >2.7 cm/m^2^		Worsening cardiac symptoms/need for cardiac transplant
Hilfiker‐Kleiner 2007^23^	Not documented	Unclear	Interventional study	*N* = 12	Effects of bromocriptine in women with SP	SP as secondary (mouse study first part)	Not documented	All women who have recovered cardiac function. Not defined.	
Hilfiker‐Kleiner 2017^24^	2005–2015	South Africa, Germany, Scotland	Retrospective cohort	*N* = 34	Outcome of SPs in PPCM patients	SP as focus	LVEF ≤45% and absence of previously known cardiomyopathy before pregnancy. States those included fulfilled PPCM criteria and references 2010 ESC position statement	Recovered LVEF ≥50% at outset of SP	
Ma'ayeh 2002^25^	2008–2019	USA	Retrospective cohort	*N* = 18	Evaluate LVEF and global longitudinal strain to predict the risk of PPCM recurrence in SP	SP as focus	LVEF <45% in third trimester and up to 5 months post‐delivery with no cause identified		For those with baseline LVEF ≤45% at start of pregnancy, recurrence defined as a reduction in LVEF of >10% in the SP/5 months post‐delivery. Otherwise, recurrence defined as per PPCM definition box
Mandal 2011^26^	2006–2009	India	Prospective cohort	*N* = 6	To study clinical profile and management of PPCM, analyze pregnancy and SP outcomes	SP as secondary	(1) Development of heart failure with no identifiable cause in the last month of pregnancy or up to 5 months postpartum; (2) no cardiac disease history; (3) LVEF <45% and/or fractional shortening <30%, and LV end‐diastolic dimension >2.7 cm/m^2^	Recovered LVEF >45%	Symptoms of heart failure. (Also inferred that the one event of maternal mortality was due to relapse)
Mishra 2006^27^	1995–2005	India	Prospective cohort	*N* = 9	To determine clinical and echocardiographic profiles of women with PPCM and ascertain the course of disease	SP as secondary	(1) Cardiac failure developing in the final month of pregnancy/first 5 months postpartum; (2) LVEF <40%; (3) no other cause found for cardiac failure		
Moulig 2019^28^	2006–2013	Germany	Prospective cohort	*N* = 16	5‐year follow‐up to determine outcomes of a PPCM cohort	SP as secondary	Diagnosis of PPCM as defined by the ESC Study Group, that is LVEF ≤45% and absence of pre‐existing heart disease	Full recovery LVEF ≥50%	Inferred that relapse would be a deterioration of LVEF in SP
Ormesher 2023^29^	2008–2020	UK and Australia	Retrospective cohort	*N* = 36	To determine prevalence of pre‐eclampsia in women with pre‐existing cardiac dysfunction	SP as focus (but PPCM population a subgroup of the wider cardiomyopathy cohort)	Not documented	All women in cohort had EF <55% (all non‐recovered)	
Pachariyanon 2023^30^	1982–2020	USA	Retrospective cohort	*N* = 45	To evaluate survival after SP in women with PPCM	SP as focus	Cardiac failure developing in last month of pregnancy/up to 5 months postpartum with no cause identified and LVEF ≤45%	Recovered LVEF ≥50%	LVEF decrease to ≤45%. In women with non‐recovered LVEF, LVEF decrease ≥10%
Pillarisetti 2014^31^	1999–2012	USA	Retrospective cohort	*N* = 35	Evaluate long‐term outcomes after PPCM	SP as secondary	Stated as PPCM but not defined further in methods		
Rajan 2023^32^	2015–2019	India	Retrospective cohort	*N* = 9	Determine predictors of outcome in women with PPCM. To determine maternal and fetal outcomes	SP as secondary	Stated as PPCM but not defined further in methods	Recovered LVEF >50%	Decrease of EF to <45%
Shah 2012^33^	2008–2011	Pakistan	Cohort	*N* = 8	Determine the course of PPCM and its risk factors	SP as secondary	Heart failure developing in the last month of pregnancy or up to 5 months postpartum, LVEF <45%, no history of pre‐existing heart disease, no other cause found for cardiac failure		Recurrence of heart failure (not defined further)
Shani 2015^34^	1993–2010	Israel	Retrospective cohort	*N* = 9	Evaluate risk factors, characteristics, and prognosis of women with PPCM in index and SPs	SP as secondary	Onset of heart failure with no cause found in the final month of pregnancy or up to 5 months postpartum, with no known cardiac disease		
Sliwa 2004^35^	1996–2001	South Africa	Prospective cohort	*N* = 6	Determine outcomes of SP in PPCM	SP as focus	Congestive heart failure that develops in the final month of pregnancy/in the first 5 months postpartum, no other cause for heart failure found, LVEF ≤40%, sinus rhythm	Non‐recovered LVEF <40% at outset of SP	Heart failure symptoms
Sinkey 2020^36^	2000–2017	USA	Retrospective cohort	*N* = 31	Examine racial disparities in outcomes of women with PPCM	SP as secondary	New cardiac failure 1month prior to birth or within 5 months postpartum with absence of underlying cause or prior cardiac issues. Left ventricular systolic dysfunction evident on echocardiogram.		
Witlin 1997^37^	1986–1994	USA	Prospective cohort	*N* = 6	Review the presentation, etiology and prognosis in PPCM	SP as secondary	(1) Onset of heart failure in the final month of pregnancy/first 5 months postpartum, (2) absence of cause for cardiac failure, and (3) absence of heart disease before final month of pregnancy		Presentation with congestive cardiac failure
Yaméogo 2018^38^	2012–2016	Burkina Faso	Prospective cohort	*N* = 29	To describe maternal and fetal outcomes after pregnancy complicated by PPCM	SP as focus	(1) Development of congestive heart failure within 1 month before delivery to 5 months postpartum, (2) no identifiable cause for heart failure, (3) LVEF <45% Note: Only includes women who were hospitalized (for any reason) in the SP but presume this is because outcomes of women who labored in a community setting would not be known.	LVEF <50% non‐recovered	Congestive heart failure

*Note*: Where the cell is in gray, this definition is not relevant to the outcomes recorded within this systematic review.

Abbreviations: EF, ejection fraction; ESC, European Society of Cardiology; FC, functional classification; LV; left ventricle/left ventricular; NYHA, New York Heart Association; PPCM, peripartum cardiomyopathy; SP, subsequent pregnancy; WHO, World Health Organization.

### 2.3 Information sources

Medline, Embase, Web of Science, and CINAHL were searched from inception until November 2023, and hand‐searching of included studies was undertaken. Authors were contacted for more information where required.

### 2.4 Search strategy

The systematic search included terms such as cardiomyopathy AND pregnancy OR peripartum cardiomyopathy OR PPCM. Searches were adapted according to the requirements of each database. Detailed search strategies for each database can be found in Appendix S1. Citations retrieved from these searches were uploaded into Covidence software. Two independent reviewers (RM, JLV, or HA) reviewed each title and abstract for inclusion with discrepancies solved by consensus of reviewers. Full texts were then retrieved for citations deemed potentially relevant. Full texts were each screened by two independent reviewers (RM, JLV, or HA) with discrepancies solved by consensus of reviewers. Digital translation software was used to determine the eligibility of studies published in a non‐English language.

### 2.5 Data extraction

Data were extracted into a pre‐designed Microsoft Excel spreadsheet by two reviewers (RM, JH, or HA) independently for each study, with disagreements solved by consensus.

Number of pregnancies or babies comprised the denominator for all outcomes, apart from death and need for cardiac transplant, where the denominator was the number of women. For the primary outcome of cardiac failure relapse, we included only studies where the outcome for the first subsequent pregnancy could be separated from any further pregnancies, and only this first subsequent pregnancy data was used in the analysis. This avoided the interaction in the likelihood of outcome occurrence when one woman had more than one subsequent pregnancy and meant that each pregnancy outcome could be seen as an independent event, giving a more accurate estimation of risk for this outcome. All other outcomes were reported from all recorded subsequent pregnancy data.

### 2.6 Assessment of risk of bias

The Joanna Briggs Institute tool for case series risk of bias assessment was used to examine the included studies, as its facets were found to be of most relevance to the included studies.^39^ Risk of bias was assessed by two reviewers independently (RM, MY). Where the answer ‘yes’ was given to less than 50% of the facets within the tool, the study was classified as a high risk of bias.

### 2.7 Data synthesis

Data were synthesized using R software version 4.3.0 and RStudio. Proportional data were meta‐analyzed with the meta package, using the metaprop command. A logit transformation of data was performed and the meta‐analysis was undertaken in a random effects model, as there was significant clinical heterogeneity evident between studies. Where appropriate data were meta‐analyzed to give pooled proportions with 95% confidence intervals. A continuity correction of 0.5 was applied in studies with zero cell frequencies. The *I* ^2^ statistic was used to assess statistical heterogeneity. As there are established problems with proportional meta‐analysis transformation models where proportions are close to zero, for outcomes where ≥70% of studies recorded zero events, a decision was made not to undertake meta‐analysis but to present the results narratively.^40^, ^41^, ^42^ Continuous data were meta‐analyzed using the metamean command. Studies were included where means and SDs were available or could be computed. Log transformed means were used to calculate a pooled mean, alongside a 95% confidence interval, in a random effects model.

## 3 RESULTS

Twenty‐nine studies were included in the analysis. In total, these studies involved 779 women with subsequent pregnancy after peripartum cardiomyopathy.^10^, ^11^, ^12^, ^13^, ^14^, ^15^, ^16^, ^17^, ^18^, ^19^, ^20^, ^21^, ^22^, ^23^, ^24^, ^25^, ^26^, ^27^, ^28^, ^29^, ^30^, ^31^, ^32^, ^33^, ^34^, ^35^, ^36^, ^37^, ^38^

### 3.1 Study selection

**FIGURE 1:**
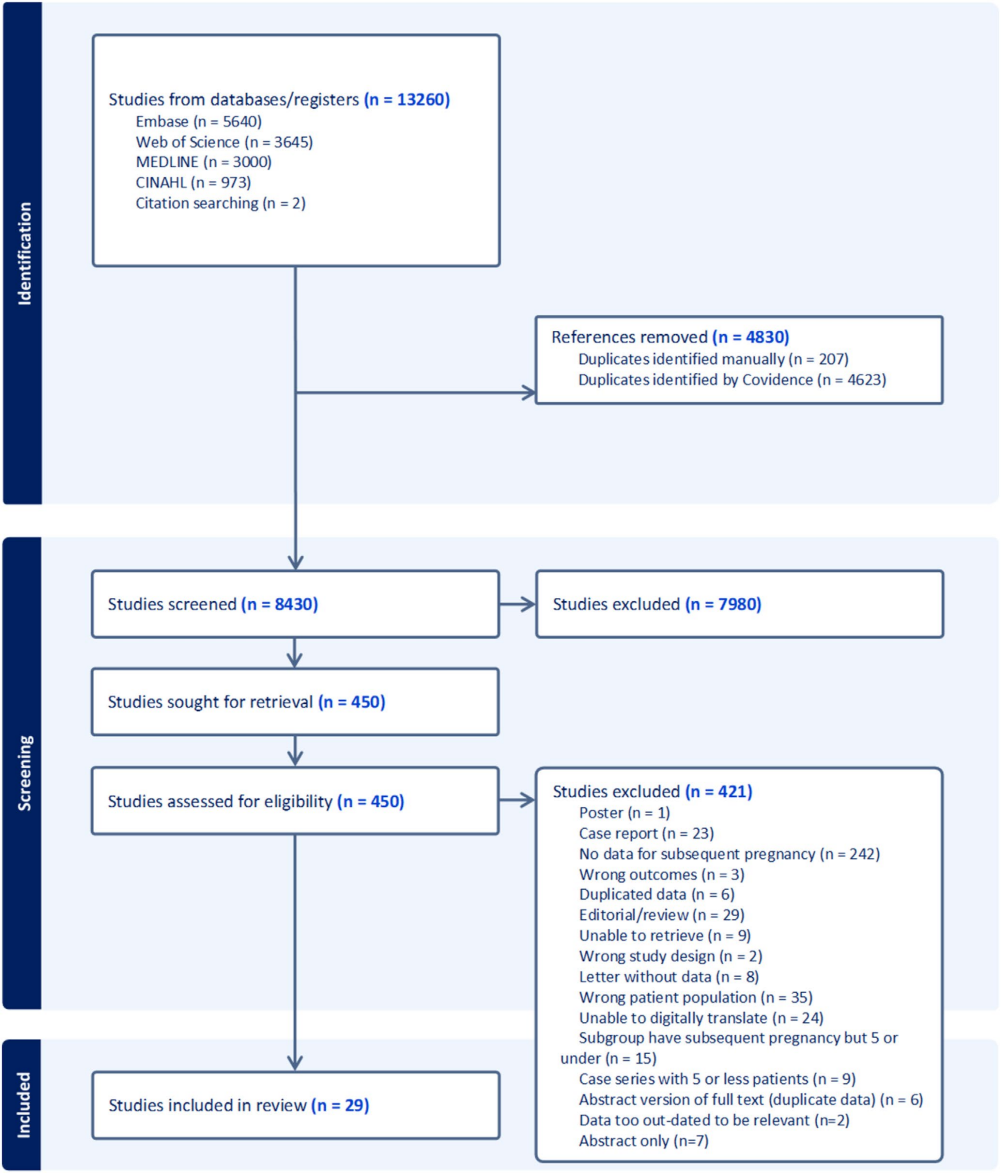
Study flow diagram.

The PRISMA flow diagram can be seen in Figure 1. We screened 8430 titles and abstracts after removal of duplicates. We sought full texts for 450 articles, and of these, 29 were included in our review. Two additional studies fitted the inclusion criteria, however the cohorts were post hoc deemed to be too dated to provide information relevant to the current clinical landscape and therefore were excluded from analysis.^43^, ^44^

### 3.2 Study characteristics

Of the 29 articles included, studies were conducted in 13 countries including the United States (12 studies), Brazil (three studies), South Africa (three studies), India (two studies), Haiti (two studies), Germany (two studies), and the UK (two studies). There was one study conducted in each of Australia, Nigeria, Denmark, Israel, Pakistan, and Burkina Faso. Four studies were conducted across more than one country. In 15 studies, the data provided on a subsequent pregnancy was a small subset of data, with the primary focus of the study being on initial outcomes within the index peripartum cardiomyopathy pregnancy. In one study, the primary results were from an animal study with a subset of data then provided on subsequent pregnancy outcomes of women where peripartum cardiomyopathy was diagnosed in a previous pregnancy. Study characteristics can be found in Table 1.

### 3.3 Risk of bias of included studies

Using the Joanna Briggs Institute risk of bias tool,^39^ five of 29 studies had a high risk of bias (<50% of ‘yes’ answers to each facet within the tool). Eighteen studies did not clearly utilize consecutive inclusion of participants. Ten studies did not have clear reporting of the demographics of the participants in the study, which was often the case where studies included subsequent pregnancy outcomes as a secondary analysis of index peripartum cardiomyopathy pregnancy outcomes. Our risk of bias assessments for each study can be found in Table S2.

### 3.4 Synthesis of results

#### 3.4.1 Primary outcomes

**FIGURE 2:**
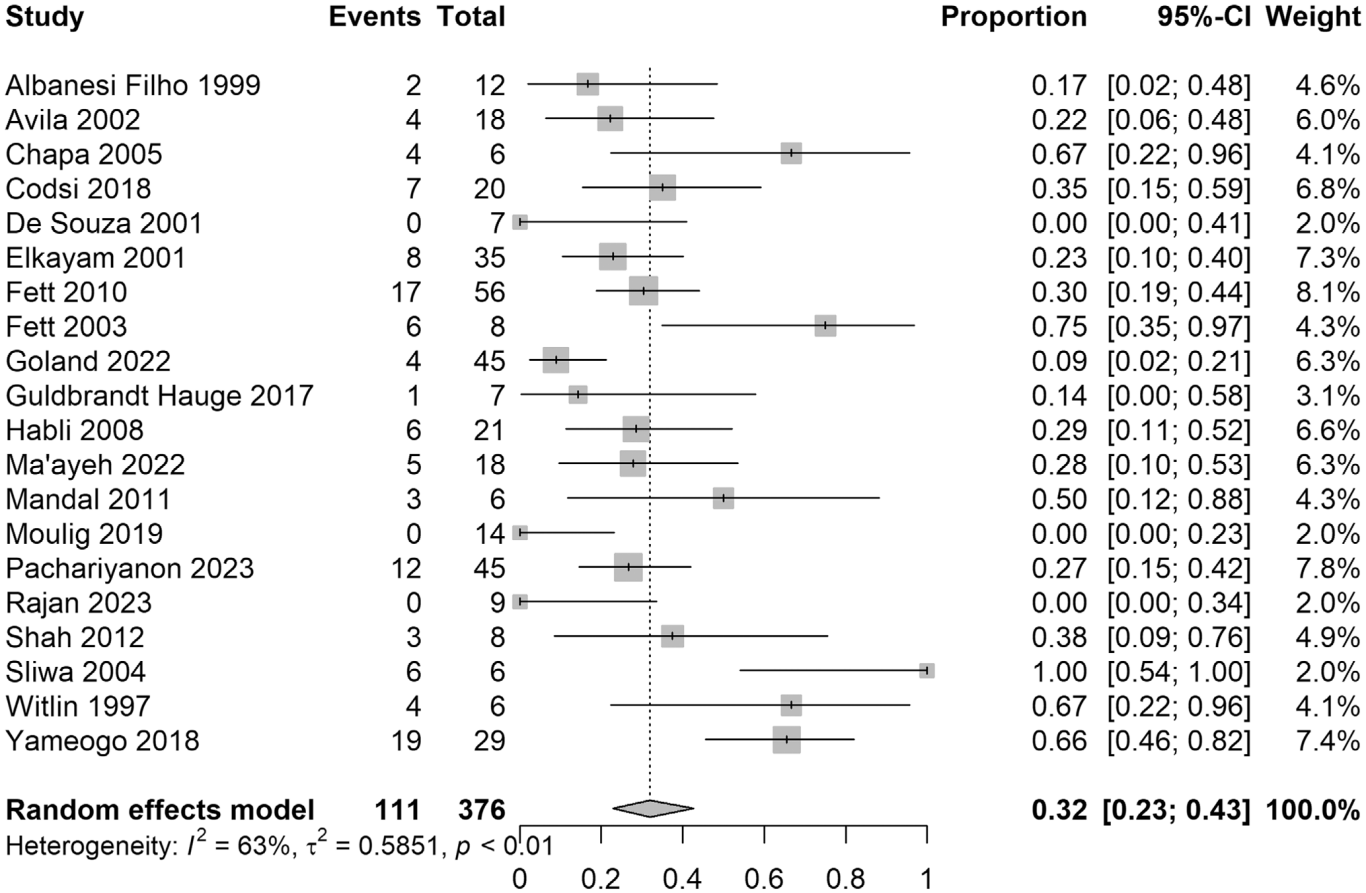
Risk of recurrence of cardiac failure in first subsequent pregnancy. Forest plot shows risk of recurrence of cardiac failure in the first subsequent pregnancy after an initial peripartum cardiomyopathy pregnancy. CI, confidence interval.

**FIGURE 3:**
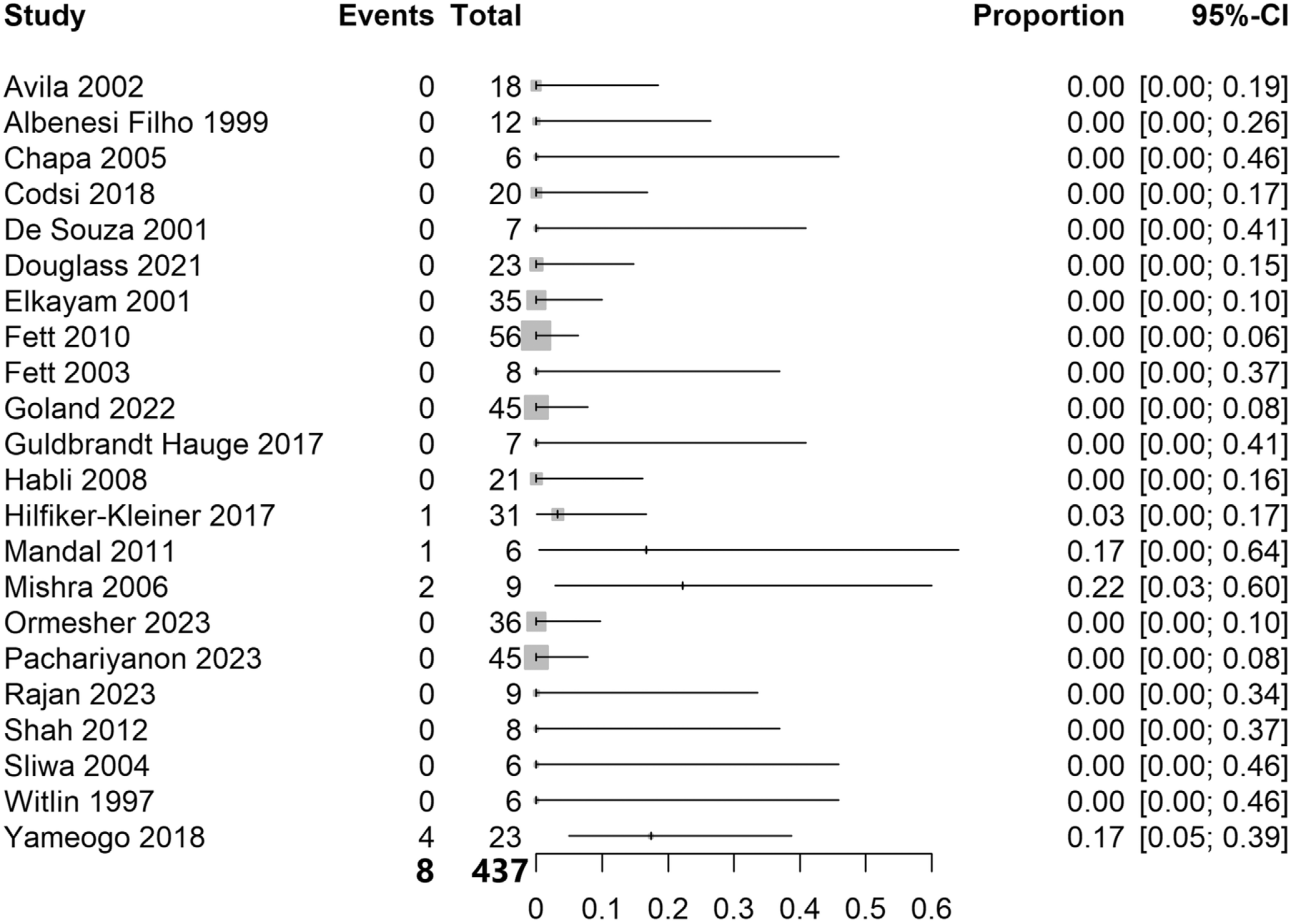
Risk of death during the course of subsequent pregnancy. Forest plot shows risk of death during the course of subsequent pregnancy after an initial peripartum cardiomyopathy pregnancy. CI, confidence interval.

For the primary outcome of relapse of cardiac failure in the first subsequent pregnancy after peripartum cardiomyopathy, 20 studies with 376 women were included in the analysis. The risk of relapse of cardiac failure was 32%, with a 95% confidence interval (CI) 0.23–0.43, *I* ^2^ = 63% (Figure 2). For the risk of death during the course of all subsequent pregnancies (not including the postpartum period), 22 studies with 437 women were included in the analysis. The majority (82%) of studies reporting the risk of death during subsequent pregnancies had zero events, and so meta‐analysis was not undertaken for this outcome. There were eight deaths in 437 women (1.8%) across the included studies (Figure 3).

#### 3.4.2 Secondary outcomes

##### Maternal outcomes

**FIGURE 4:**
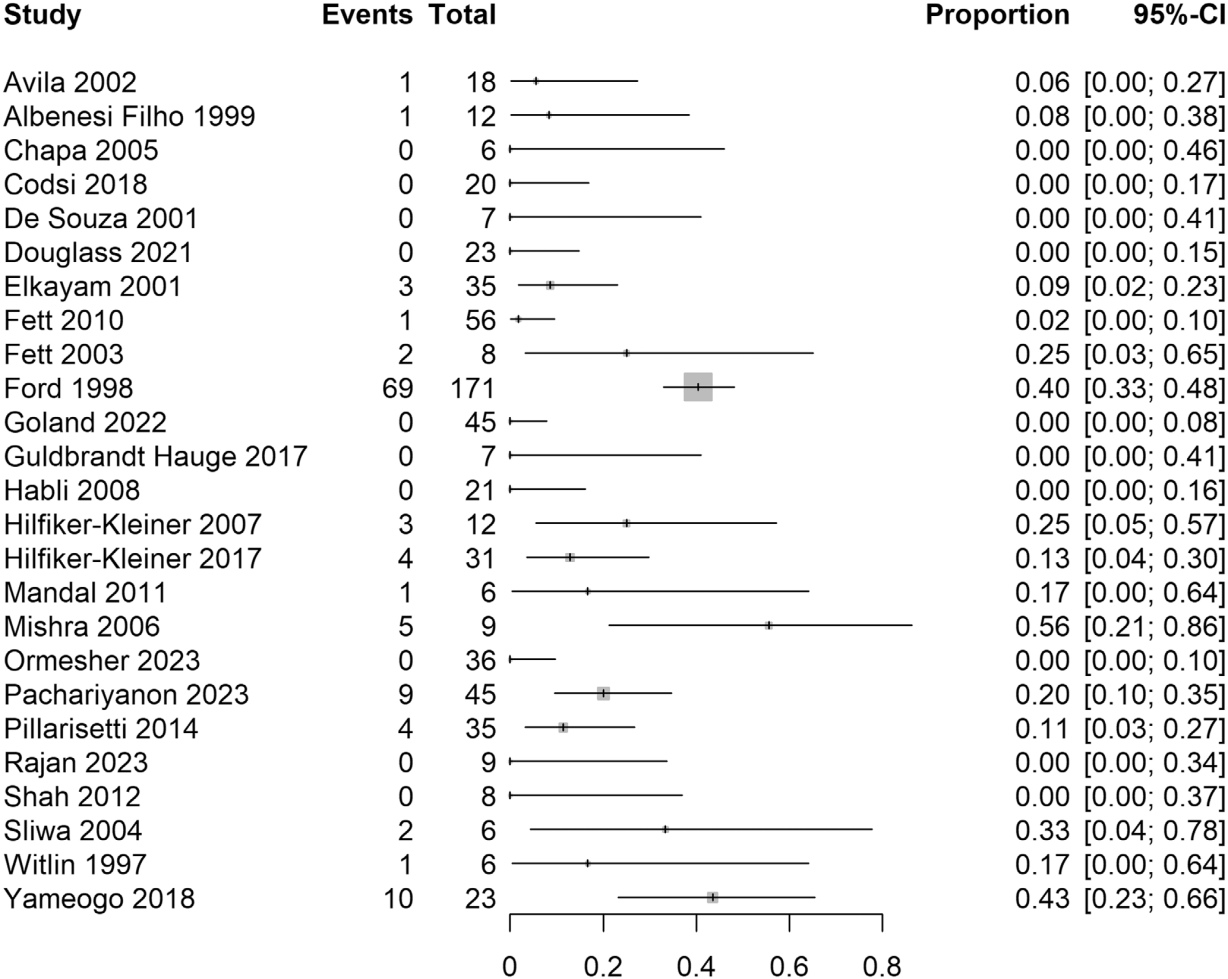
Risk of death at any time point during or after subsequent pregnancy. Forest plot shows the risk of death at any time point during or after subsequent pregnancy in women with previous peripartum cardiomyopathy. CI, confidence interval.

**FIGURE 5:**
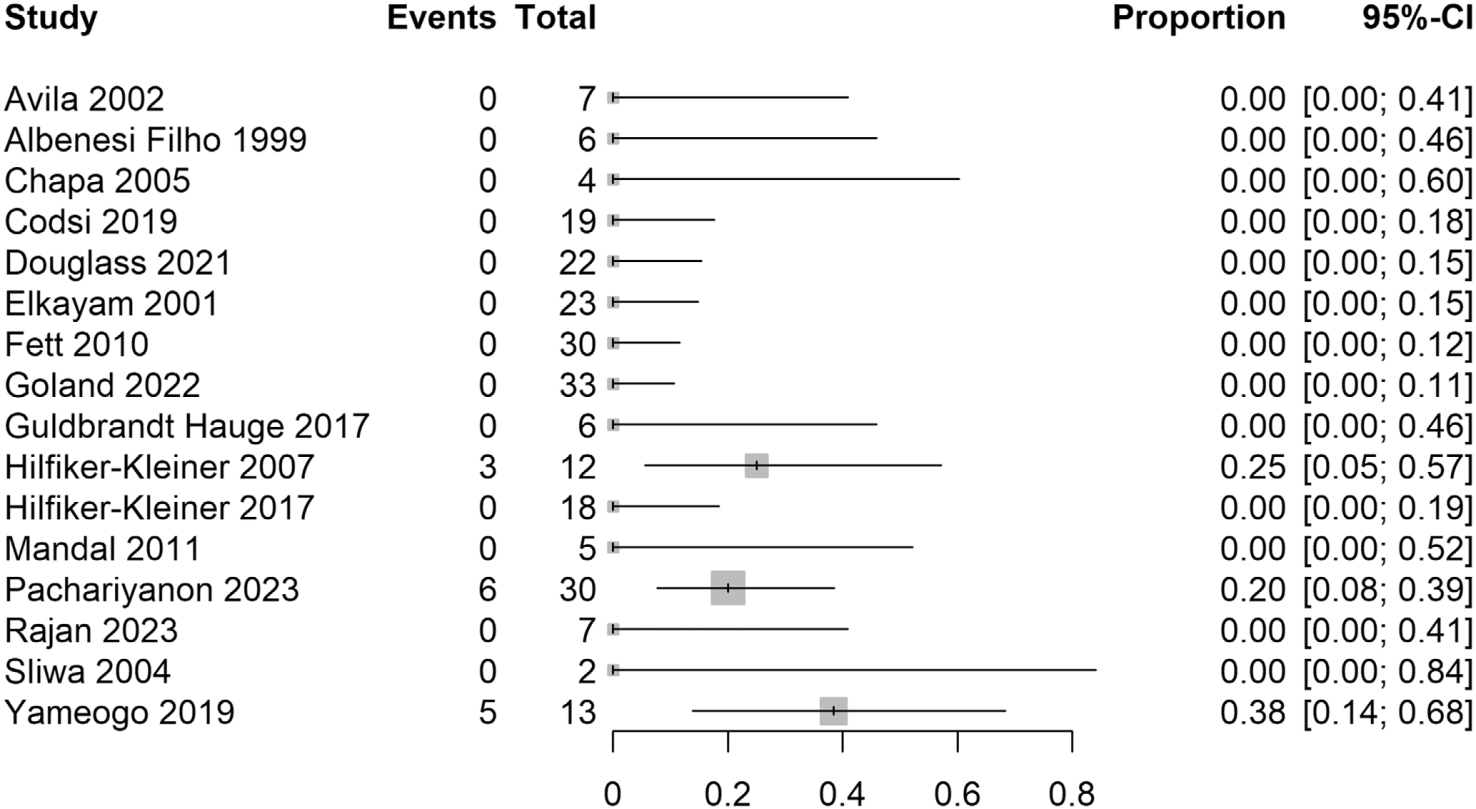
Risk of death at any time point during or after subsequent pregnancy with recovered cardiac function at pregnancy outset. Forest plot shows risk of death at any time point during or after subsequent pregnancy in women with previous peripartum cardiomyopathy, with recovered cardiac function at pregnancy outset. CI, confidence interval.

**FIGURE 6:**
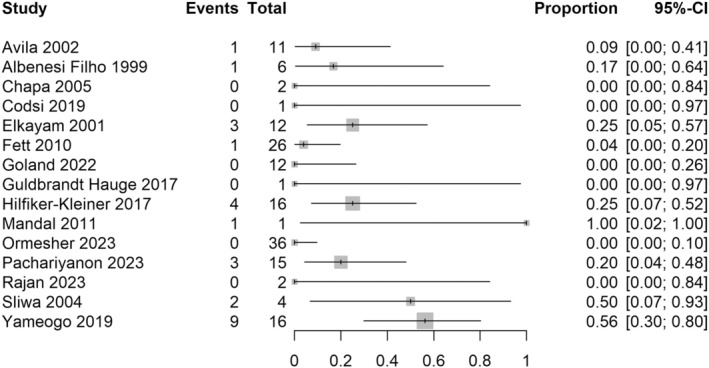
Risk of death at any time point during or after subsequent pregnancy with non‐recovered cardiac function at pregnancy outset. Forest plot shows risk of death at any time point during or after subsequent pregnancy in women with previous peripartum cardiomyopathy, with non‐recovered cardiac function at pregnancy outset. CI, confidence interval.

For the outcome of relapse of cardiac failure in the first subsequent pregnancy in those with recovered (11 studies, 123 pregnancies) and non‐recovered (10 studies, 55 pregnancies) cardiac function, the risks were 24%, 95% CI 0.16–0.35, *I* ^2^ = 39% and 36%, 95% CI 0.24–0.50, *I* ^2^ = 0%, respectively. Most commonly, authors used a definition of recovered cardiac function to be an ejection fraction over 45%–55% at pregnancy outset (see Table 1). For the outcome of death during or after subsequent pregnancy, we were unable to create a pooled estimate as studies followed up women for different amounts of time post‐delivery, with most studies not providing the total length of follow‐up. Forest plots are therefore included (Figures 4, 5, 6) to provide a graphical display of study results for the outcome of death and death by recovered and non‐recovered cardiac function at pregnancy outset, without a pooled estimate. The risk of being listed for cardiac transplant during a subsequent pregnancy was only clearly recorded in three small studies (43 women) with a pooled risk of 10%, 95% CI 0.04–0.23, *I* ^2^ = 0%.

The risk of postpartum hemorrhage in a subsequent pregnancy (three studies, 64 pregnancies) was 17%, 95% CI 0.06–0.39, I^2^ = 56%. For the outcomes of pregnancy‐induced hypertension (three studies, 46 pregnancies), pre‐eclampsia (seven studies, 132 pregnancies) and hypertensive disorder composite (six studies, 160 pregnancies), the risks of developing these during a subsequent pregnancy were 8%, 95% CI 0.03–0.20, *I* ^2^ = 0%; 7%, 95% CI 0.03–0.17, *I* ^2^ = 17% and 12%, 95% CI 0.06–0.22, *I* ^2^ = 47%, respectively.

##### Fetal and neonatal outcomes

The risk of preterm birth in a subsequent pregnancy at <37 weeks (12 studies, 212 pregnancies) and <34 weeks (nine studies, 132 pregnancies) was 22%, 95% CI 0.17–0.29, *I* ^2^ = 5% and 8%, 95% CI 0.04–0.15, *I* ^2^ = 0%, respectively. The majority (78%) of studies reporting the risk of preterm birth <28 weeks had zero events, and so meta‐analysis was not undertaken for this outcome. There were two events of preterm birth <28 weeks in 132 pregnancies (1.5%), across the nine studies included. Risks of stillbirth in a subsequent pregnancy (14 studies, 368 babies) or neonatal death (six studies, 143 babies) were not meta‐analyzed for the same reason, however there were three stillbirths in 368 babies (0.8%) and four neonatal deaths in 143 babies (2.8%).

For the outcome of fetal growth restriction (five studies, 110 babies), the risk in a subsequent pregnancy was 9%, 95% CI 0.04–0.18, *I* ^2^ = 0%. For the outcome of small for gestational age, meta‐analysis was not performed as only two studies explicitly recorded this outcome, with eight events recorded in 46 babies (17.4%). For the outcome of birthweight among all subsequent pregnancies (six studies, 153 babies), the mean birthweight was 2921 g, 95% CI 2634–3240 g, *I* ^2^ = 93%. The mean gestational age at birth for all subsequent pregnancies (six studies, 143 pregnancies) was 38.0 weeks, 95% CI 37.6–38.4 weeks, *I* ^2^ = 29%. Forest plots for secondary outcomes not included in the main body of this review can be found in Appendix S3.

#### 3.4.3 Subgroup analyses

**FIGURE 7:**
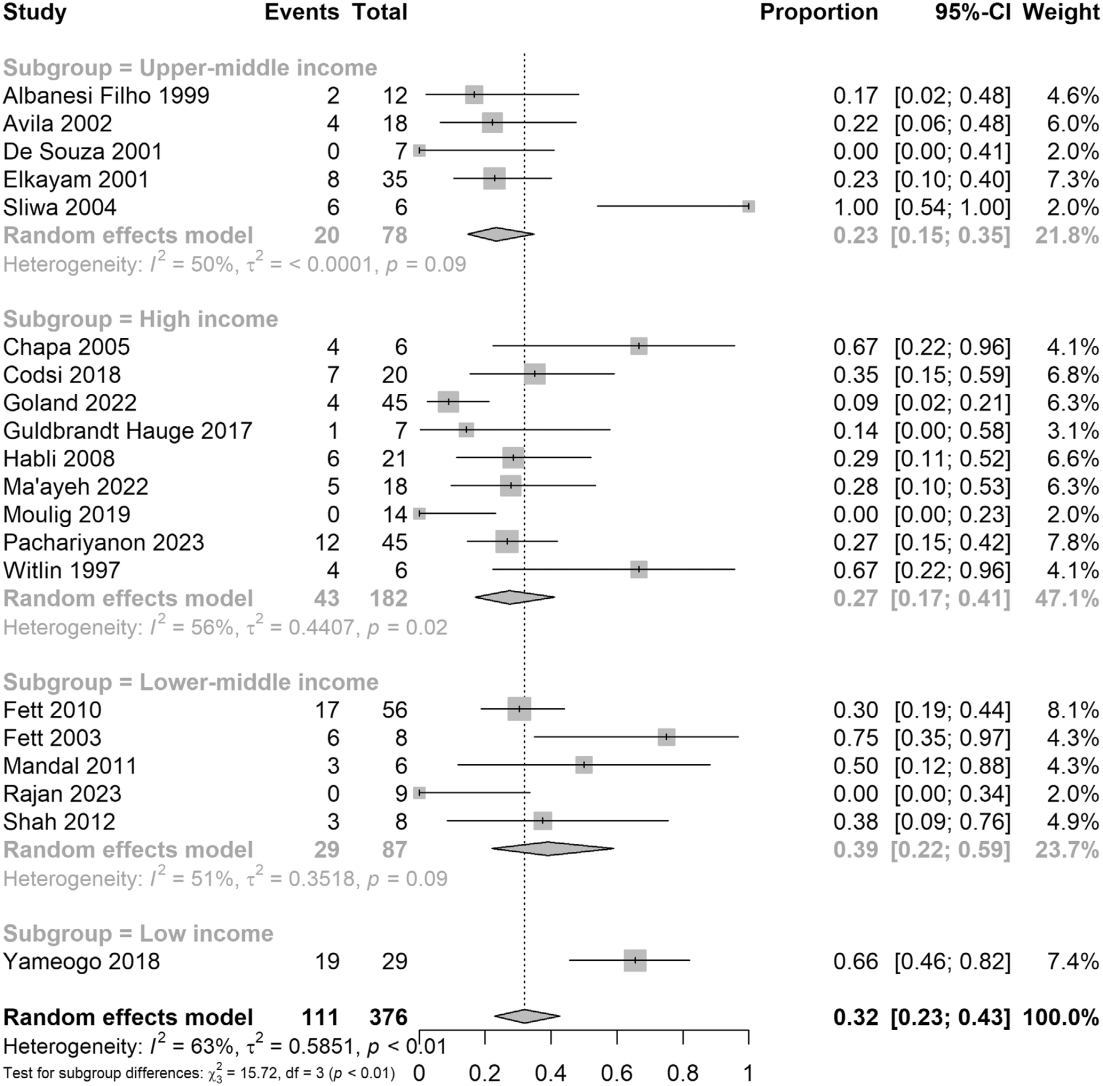
Risk of recurrence of cardiac failure in the first subsequent pregnancy with studies in subgroups by World Bank Income Status. Forest plot shows risk of recurrence of cardiac failure in the first subsequent pregnancy after an initial peripartum cardiomyopathy pregnancy, with subgroups by World Bank Income status of the country in which the study was conducted. CI, confidence interval.

For the primary outcome of relapse of cardiac failure in the first subsequent pregnancy, this was analyzed according to the World Bank Income Level of the country in which each study was conducted. Nine studies for this outcome were conducted in a high‐income setting (182 women), five in an upper‐middle income setting (78 women), five in a lower‐middle income setting (87 women), and one in a low‐income setting (29 women). For high, upper‐middle, lower‐middle, and low‐income countries, respectively, the risk of cardiac failure relapse in the first subsequent pregnancy was 27%, 95% CI 0.17–0.41; 23%, 95% CI 0.15–0.35; 39%, 95% CI 0.22–0.59; and 66%, 95% CI 0.46–0.82 (*p* = <0.01 for subgroup differences) (Figure 7).

## 4 DISCUSSION

Here we provide a summary of existing studies that provide data on outcomes in subsequent pregnancies, following a diagnosis of peripartum cardiomyopathy in an index pregnancy. This provides evidence on which to base information given to women and to subsequently direct counseling and advice. Notably, the risk overall of a relapse in the first subsequent pregnancy was pooled at 32%, with this figure increasing to 36% where cardiac function has not recovered at the outset of the subsequent pregnancy and reducing to 24% where cardiac function has recovered. Risks clearly remain high for both groups, however considering differences in outcome with varying cardiac function at subsequent pregnancy outset may help with individualizing discussions. While the meta‐analysis of mortality data was limited by the fact that most authors did not report the length of follow‐up for their cohort, just during the course of a subsequent pregnancy, there were eight deaths among 437 women. In individual studies recording death at any time point during or after the subsequent pregnancy, the risk of death during the study period ranged greatly, from 0% to 56% of the cohort.^13^, ^27^

While this study clearly highlights the substantial maternal risks, it is also vital women are provided with information on the likelihood of an adverse fetal outcome with a subsequent pregnancy. Although we could not distinguish between spontaneous and iatrogenic preterm birth, which would be imperative for any future observational studies to record, rates of preterm birth were high within these cohorts. Our results demonstrate an 8% risk of preterm birth <34 weeks—given the established higher burden of morbidity and mortality in this group, this is an important finding for women and clinicians to be aware of.^45^, ^46^

Disparate outcomes for women, depending on the income status of their country, highlight the stark healthcare inequalities that are faced globally. While we were only able to include data from one low‐income country, Burkina Faso, the outcomes were exceptionally poor in this study.^38^ A significant limitation of this study was that it only included women who presented to hospital at any point during their subsequent pregnancy and, as authors speculate, there may have been women with a previous diagnosis of peripartum cardiomyopathy with uneventful subsequent pregnancies, who consequently did not present to hospital during their pregnancy or labor. While being mindful of its limitations, it was important to include this study in order to provide a global perspective on subsequent pregnancy outcomes after peripartum cardiomyopathy. As we were only able to include data from one low‐income country, further caution must be taken in the interpretation of the results from this subgroup—it is likely that the estimate may be different if there were studies from an increasing number of low‐income countries eligible for inclusion.

By searching multiple databases with broad search terms and no limitations on study language, this systematic review provides an up‐to‐date, comprehensive summary of existing evidence for subsequent pregnancy outcomes after peripartum cardiomyopathy. To our knowledge, there is only one existing systematic review on this subject, which includes 18 studies, with meta‐analysis restricted to pairwise data and limited outcomes included.^8^ As well as the additional published data we included in our review in comparison to the existing systematic review, we also include a subset of peripartum cardiomyopathy data from the retrospective cohort study by Ormesher et al.; a study which determined the prevalence of pre‐eclampsia and other adverse pregnancy outcomes among women with known cardiomyopathy in the UK and Australia.^29^ The outcomes for just those women with peripartum cardiomyopathy are not available in the original study, however we were able to obtain this data for our systematic review through contacting the study authors. We therefore build significantly on the previous review by providing both increased breadth and depth to the current knowledge base and helping to fill an evidence gap that is highlighted in the 2018 European Society of Cardiology (ESC) Guidelines on cardiovascular disease in pregnancy.^47^ We included a broad range of maternal and fetal outcomes, most of which were not included in the existing review, aiming to encompass outcomes important to both women and clinicians.

The aim of subgroup analysis by country income status was to provide population‐specific outcome data. Given the paucity of low‐income country studies, caution must be taken in the interpretation of the results—as there were small numbers of studies and women eligible for inclusion from the outset, statistical power may not be optimal to find truly significant differences between groups. Analysis of the recurrence of cardiac failure in the subsequent pregnancy, based on recovered and non‐recovered cardiac function at the outset of the subsequent pregnancy, also provides useful outcome data to provide a more accurate estimation of risk to an individual.

This systematic review and meta‐analysis were limited by reporting in primary studies. While we attempted to remove outcome data from women who underwent a termination of pregnancy or a miscarriage during their subsequent pregnancy, some studies did not document whether any of their cohort underwent a termination of pregnancy or miscarriage, or the data could not be separated. This is an important caveat when interpreting and applying the results of this study, as there may be slight variations in the true risks associated with subsequent pregnancies. The decision to exclude miscarriage as an outcome and where possible not to include this group of women in analysis was to avoid an underestimation of risks for women who have ongoing pregnancies. However, consideration must be given to the fact that a continuum exists whereby the etiology underlying a late miscarriage may be similar to that of an extreme preterm birth,^48^ with potentially relevant information being omitted from this systematic review by excluding the former group.

The definitions used by authors to determine an initial diagnosis of peripartum cardiomyopathy also varied somewhat between studies, as did definitions of relapse in the subsequent pregnancy. These different definitions are recorded in our study characteristics table for clarity. Additionally, the definitions of secondary outcomes varied between authors, and for many of the outcomes, including postpartum hemorrhage, pre‐eclampsia, and pregnancy‐induced hypertension, the majority of included studies did not provide definitions, limiting the applicability of our findings. For many of the secondary outcomes, there were few studies eligible for inclusion, limiting the precision of pooled estimates. Even where a larger number of studies were available for an outcome, the numbers of women included in these studies were mostly small, further limiting the precision of estimates and the generalizability of our findings. Furthermore, while two very dated studies were excluded post hoc that met the original inclusion criteria, there remain limited cohorts providing truly contemporary data, and therefore, this must be taken into account when interpreting and applying the results of this review. Over time, there have been significant changes to patient demographics, diagnosis, and management of peripartum cardiomyopathy, and so outcomes are likely to change over time as a reflection of this.

There is also the potential for both publication and reporting bias in the included studies. Where potential authors have a cohort with poor outcomes, it is possible they may be less likely to publish and publicize those outcomes. In contrast, the opposite may be true in that where women have positive outcomes in subsequent pregnancies, this may not have been deemed significant and therefore not recorded or published. Many of the included patient series are small and there is the potential that if an outcome did not occur, then authors did not report it in their published work, therefore leading to an artificial over‐inflation of risks. It must also be considered that our results are based on women who became pregnant and continued their pregnancy after an initial peripartum cardiomyopathy diagnosis—women with more severe disease may be less likely to become pregnant again or to continue the pregnancy. In women with more severe disease, it is therefore harder to ascertain the true risks, as numbers pursuing another pregnancy are smaller. The existing ESC guideline for the management of cardiovascular diseases during pregnancy states that where ejection fraction has not recovered to >50%–55%, a subsequent pregnancy should be discouraged in women with peripartum cardiomyopathy diagnosed in a previous pregnancy.^47^ While the findings of this review are in keeping with this, as we found a higher risk in those with non‐recovered cardiac function, we also find a significant risk of relapse in those with recovered cardiac function.

## 5 CONCLUSION

Subsequent pregnancy after peripartum cardiomyopathy poses significant maternal and fetal risks, with this study providing quantification of key outcomes. There is a notable lack of published studies on this subject from countries outside the United States. We are aware of the ongoing UKOSS (UK Obstetric Surveillance System) study, exploring the outcomes of women in subsequent pregnancies with a prior cardiomyopathy diagnosis, which may help to provide more UK data. Increasing the number of robust published studies on this subject with clearly defined outcomes and eligibility criteria would be vital to advance understanding. Notwithstanding study limitations, we provide a valuable source of evidence on subsequent pregnancy outcomes after peripartum cardiomyopathy.

## AUTHOR CONTRIBUTIONS

**Rebecca Man**: conceptualization, methodology, data curation, analysis, writing: original, writing: review and editing. **Jack Le Vance and Hafsa Abdullahi**: data curation, investigation, writing: review and editing. **Clare Davenport**: formal analysis, investigation, methodology, data curation. **Mengshi Yuan**: data curation, investigation. **Laura Ormesher**: data curation. **Sana Usman**: data curation, writing: review and editing. **James Castleman and Caroline Fox**: writing: review and editing. **Lucy Hudsmith and R. Katie Morris**: supervision. **Victoria Hodgetts Morton**: formal analysis, supervision.

## CONFLICT OF INTEREST STATEMENT

The authors report no conflicts of interest.

## Supporting information


Data S1.
