# Supplementary material for: Maternal and fetal outcomes in subsequent pregnancies after peripartum cardiomyopathy: A systematic review and meta‐analysis
Source: Acta Obstet Gynecol Scand. 2025 Apr 30;104(6):1009–25. doi: 10.1111/aogs.15117 (PMC12087507; doi:10.1111/aogs.15117)
Supplement: Supplementary file 1 — Data S1. [file AOGS-104-1009-s001.docx]

**Appendix S1: Systematic review search terms**

A: Medline search 14/11/23


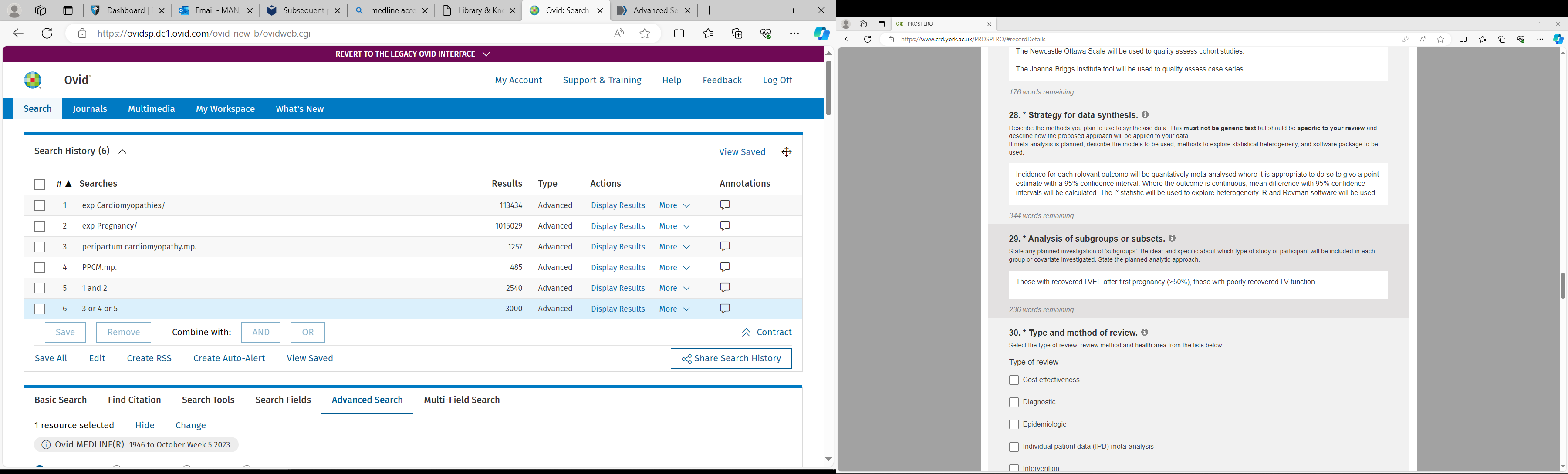


B: Embase search 14/11/23


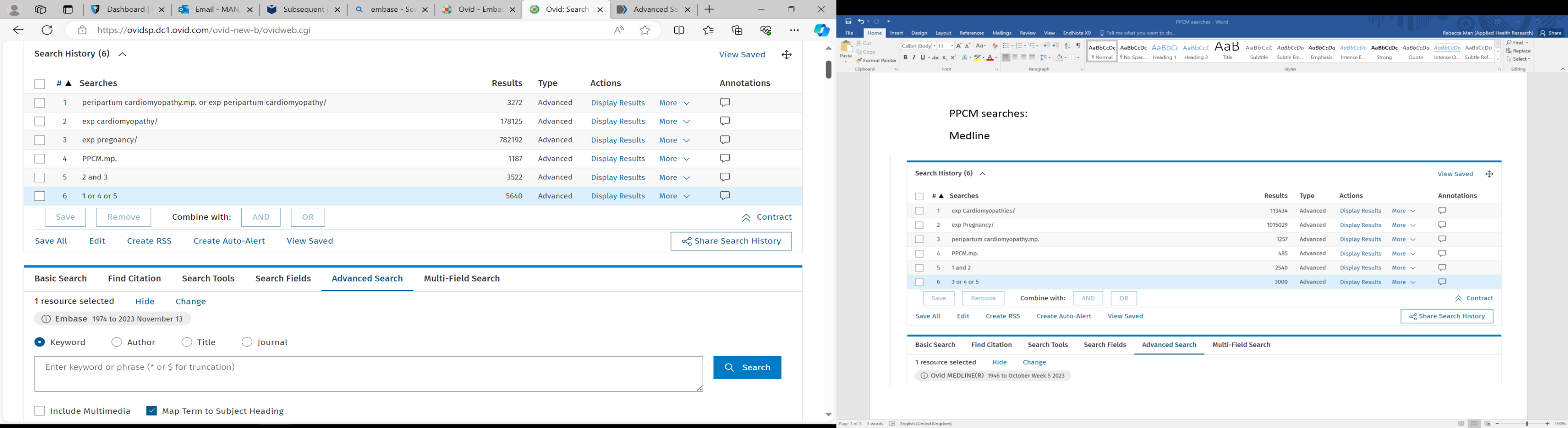


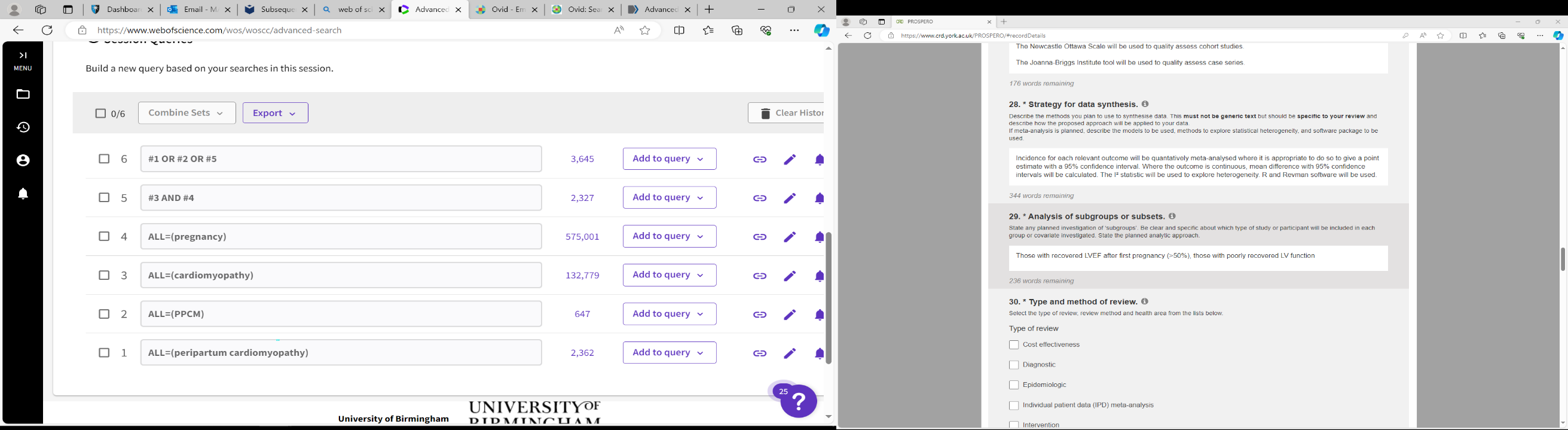
C: Web of Science search 14/11/23

D: CINAHL via EBSCO search 14/11/23


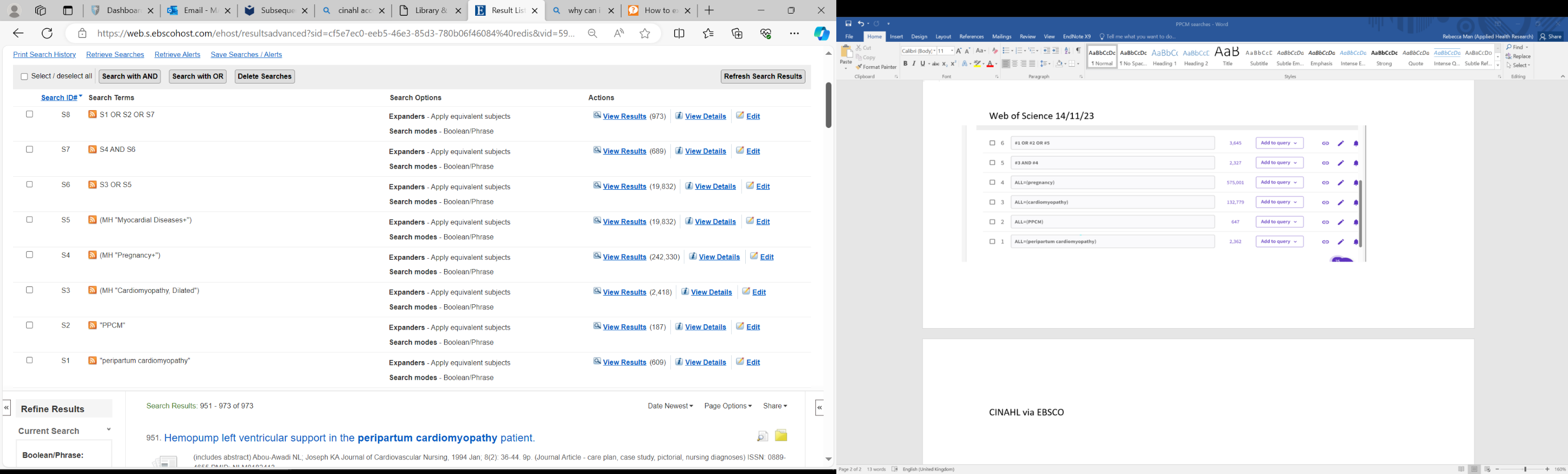


**Table S2: risk of bias assessments for each included study.**

| **Author, year** | **Clear criteria for inclusion of participants?** | **Was the condition measured in a standard, reliable**  **way for all participants included?** | **Were valid methods used for identification of the condition for all participants included?** | **Were participants included consecutively?** | **Was there complete inclusion of participants?** | **Was there clear reporting of the demographics of the participants in the study?** | **Was there clear reporting of clinical information of the participants?** | **Were the outcomes or follow up results of cases clearly reported?** | **Was there clear reporting of the presenting site(s)/clinic(s) demographic information?** | **Was statistical analysis appropriate?** | **Overall appraisal** |
| --- | --- | --- | --- | --- | --- | --- | --- | --- | --- | --- | --- |
| Albanesi Filho 1999 | Yes | Yes | Yes | Unclear | Unclear | Yes | Yes | Yes | No | N/A | Include |
| Avila 2002 | Yes | Yes | Yes | Unclear | Unclear | No | Yes | Yes | No | Yes | Include |
| Chapa  2005 | Yes | Yes | Yes | Yes | Yes | No | Yes | Yes | No | N/A | Include |
| Codsi  2018 | Yes | Yes | Yes | Yes | Yes | Yes | Yes | Yes | Yes | Yes | Include |
| De Souza 2001 | Unclear | Unclear | Unclear | Yes | No | No | Yes | Yes | No | Yes | Include |
| Douglass 2021 | Yes | Yes | Yes | Yes | Yes | No | Yes | Yes | Yes | Yes | Include |
| Elkayam 2001 | Yes | Yes | Yes | No | No | Yes | Yes | Yes | No | Yes | Include |
| Fett 2010 | Yes | Yes | Yes | No | No | No | No | No | No | Yes | Include |
| Fett 2003 | No | Unclear | Unclear | Unclear | No | No | No | Yes | Yes | Yes | Include |
| Ford 1998 | Yes | Yes | Yes | Unclear | No | No | No | No | No | Yes | Include |
| Goland 2022 | Yes | Yes | Yes | Unclear | Unclear | Yes | Yes | Yes | No | Yes | Include |
| Guldbrandt Hauge 2017 | Yes | Yes | Yes | Yes | Yes | Yes | Yes | Yes | No | Yes | Include |
| Habli 2008 | Yes | Yes | Yes | Yes | Yes | Yes | Yes | Yes | No | Yes | Include |
| Hilfiker-Kleiner 2017 | Yes | Yes | Yes | Unclear | No | Yes | Yes | Yes | Yes | Yes | Include |
| Hilfiker-Kleiner 2007 | Yes | Unclear | Yes | Unclear | Unclear | No | No | Yes | No | Yes | Include |
| Ma’ayeh 2022 | Yes | Yes | Yes | Unclear | Unclear | Yes | Yes | Yes | Yes | Yes | Include |
| Mandal 2011 | Yes | Yes | Yes | Yes | Yes | No | Yes | Yes | No | N/A | Include |
| Mishra  2006 | Yes | Yes | Yes | Yes | No | Yes | Yes | Yes | No | Yes | Include |
| Moulig 2019 | Yes | Yes | Yes | Yes | No | Yes | Yes | Yes | No | Yes | Include |
| Ormesher 2023 | No (for PPCM subgroup not clear) | Yes | Yes | Unclear | Unclear | No (not for PPCM subgroup) | Yes | Yes | No (not for PPCM subgroup) | Yes | Include |
| Pachariyanon 2023 | Yes | Yes | Yes | Unclear | Unclear | Yes | Yes | Yes | Yes | Yes | Include |
| Pillarisetti 2014 | Yes | Yes | Yes | Yes | Yes | Yes | Yes | Yes | Yes | Yes | Include |
| Rajan 2023 | Unclear | Unclear | Yes | Unclear | Unclear | Yes | Yes | Yes | Yes | Yes | Include |
| Shah 2012 | Yes | Yes | Yes | Unclear | No | Yes | Yes | Yes | No | Yes | Include |
| Shani 2015 | Yes | Yes | Yes | Yes | Yes | Yes | Yes | Yes | Yes | Yes | Include |
| Sliwa 2004 | Yes | Yes | Yes | Unclear | Unclear | Yes | Yes | Yes | No | Yes | Include |
| Sinkey 2020 | Yes | Yes | Yes | Unclear | No | Yes | Yes | Yes | Yes | Yes | Include |
| Witlin 1997 | No | Yes | Yes | Unclear | Unclear | Yes | Yes | Yes | No | Yes | Include |
| Yameogo 2018 | Yes | Yes | Yes | Unclear | No | Yes | Yes | Yes | No | Yes | Include |

**Appendix S3: additional forest plots showing outcomes in subsequent pregnancies after an index peripartum cardiomyopathy pregnancy.** Abbreviations: CI- confidence interval, SD- standard deviation.


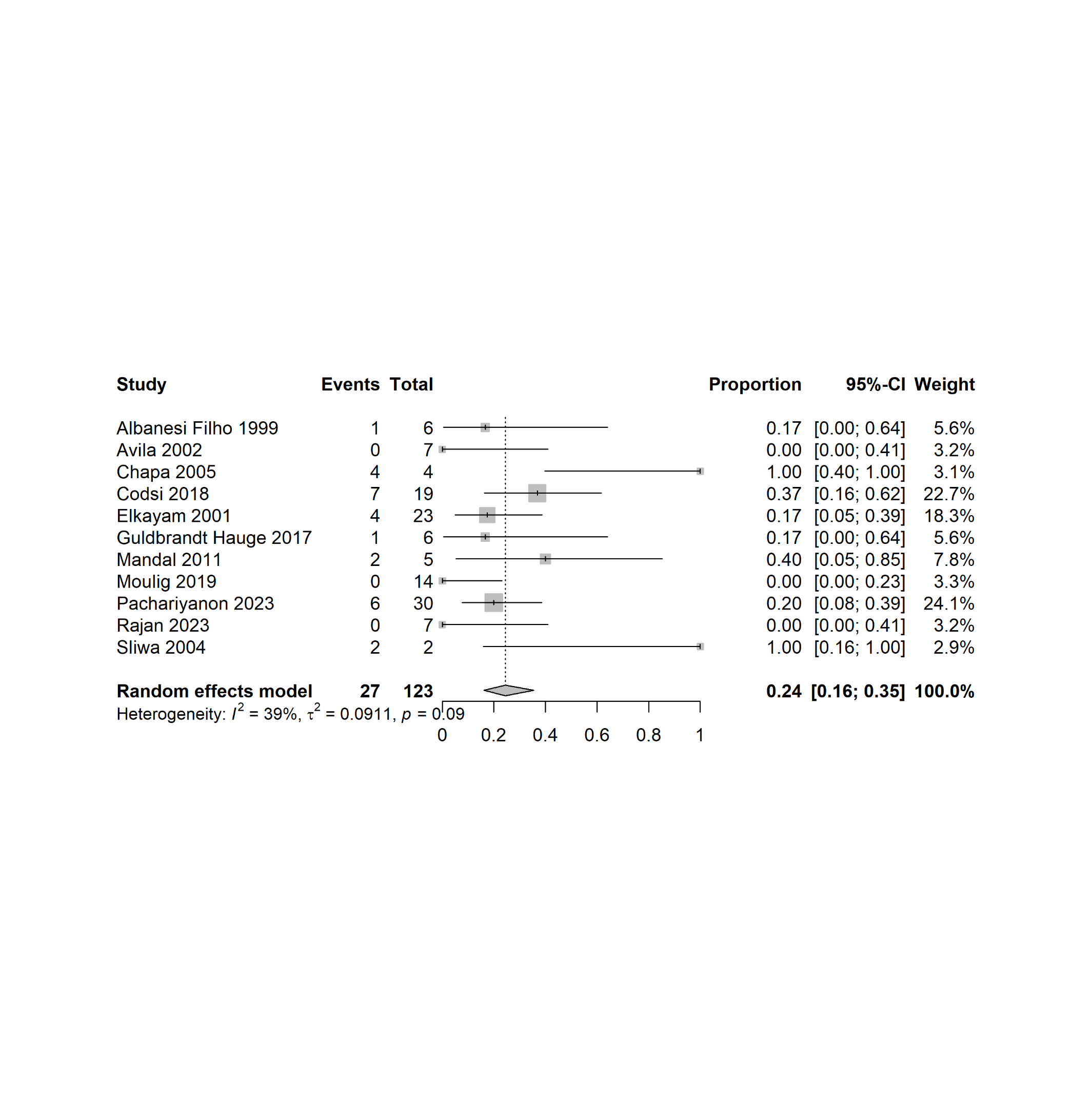


Figure S3a: Forest plot to show the proportion of women who experienced a relapse of cardiac failure in the first subsequent pregnancy after peripartum cardiomyopathy in those with recovered cardiac function.


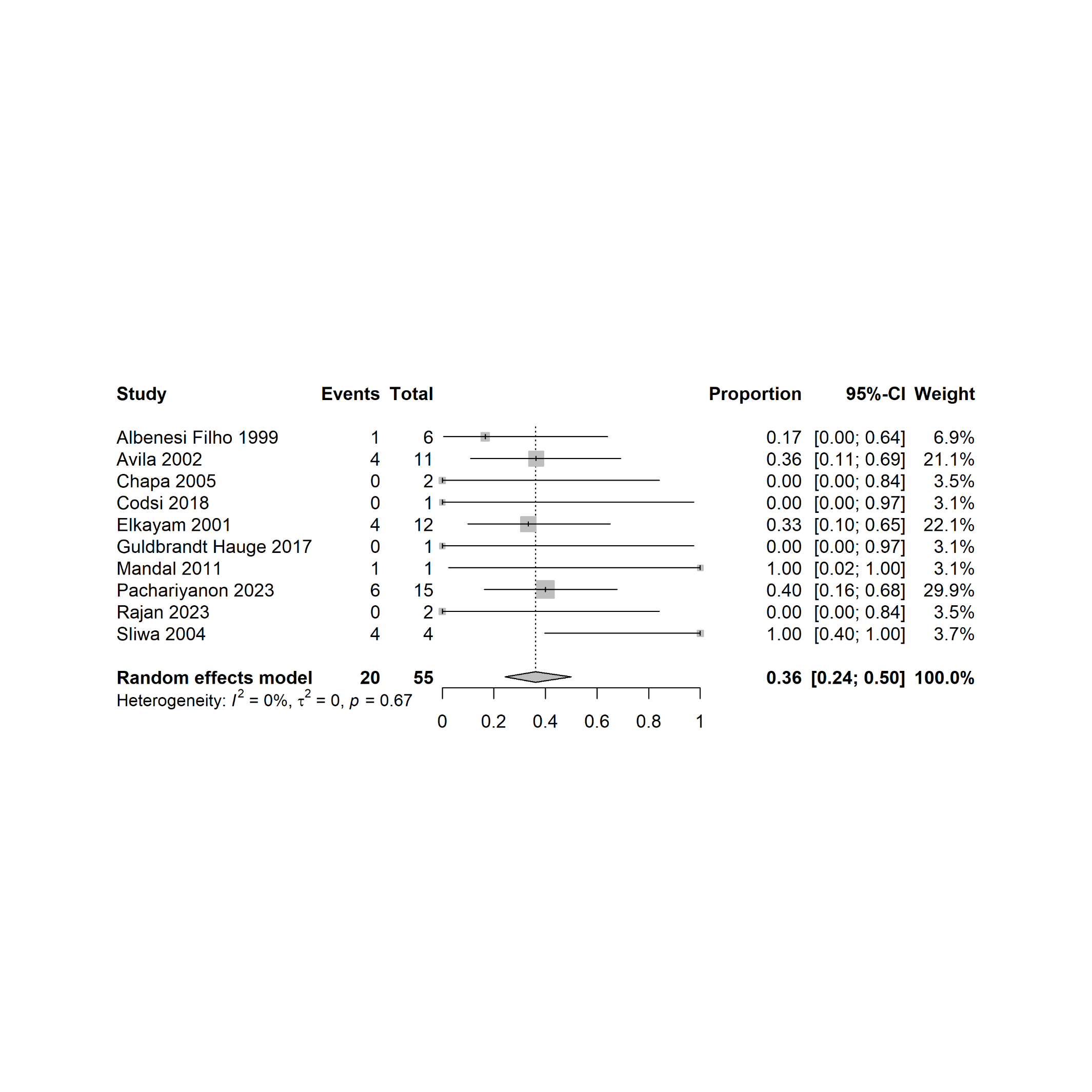


Figure S3b: Forest plot to show the proportion of women who experienced a relapse of cardiac failure in the first subsequent pregnancy after peripartum cardiomyopathy in those with non-recovered cardiac function


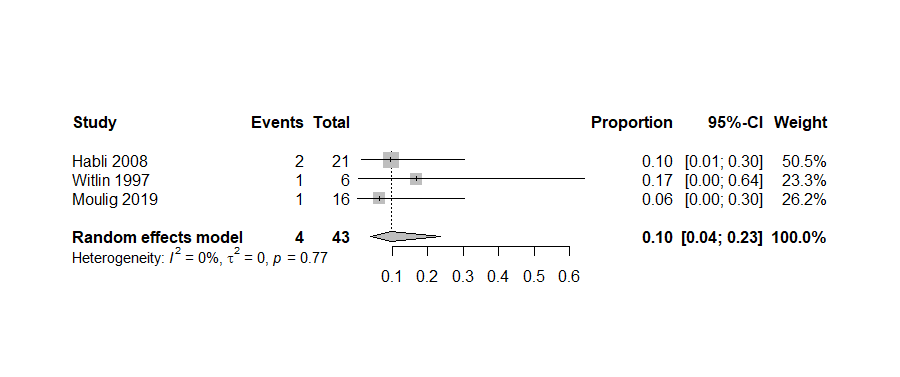


Figure S3c: Forest plot to show the proportion of women being listed for cardiac transplant in subsequent pregnancies after peripartum cardiomyopathy


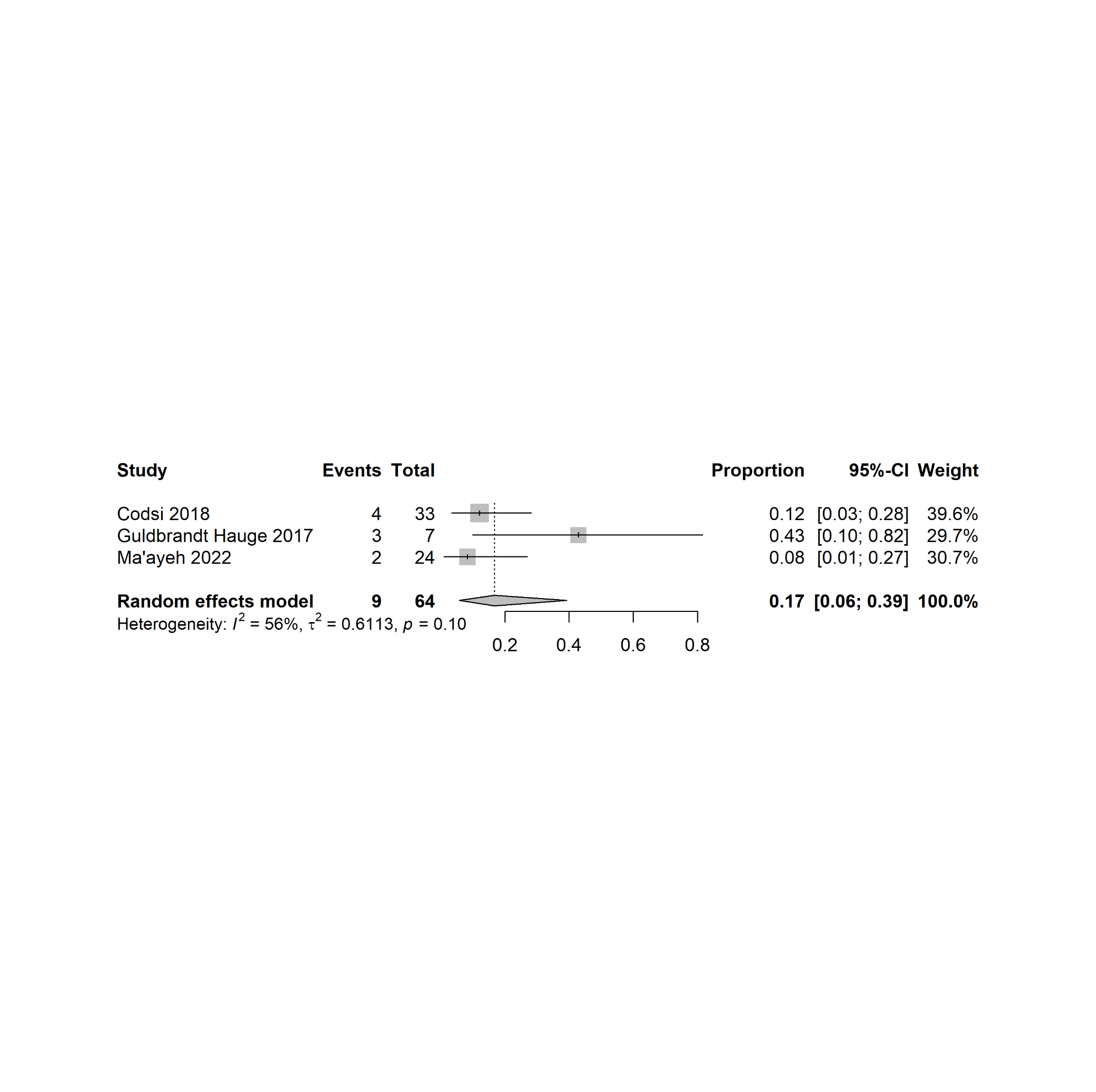


Figure S3d: Forest plot to show the proportion of births resulting in postpartum haemorrhage in subsequent pregnancies after peripartum cardiomyopathy


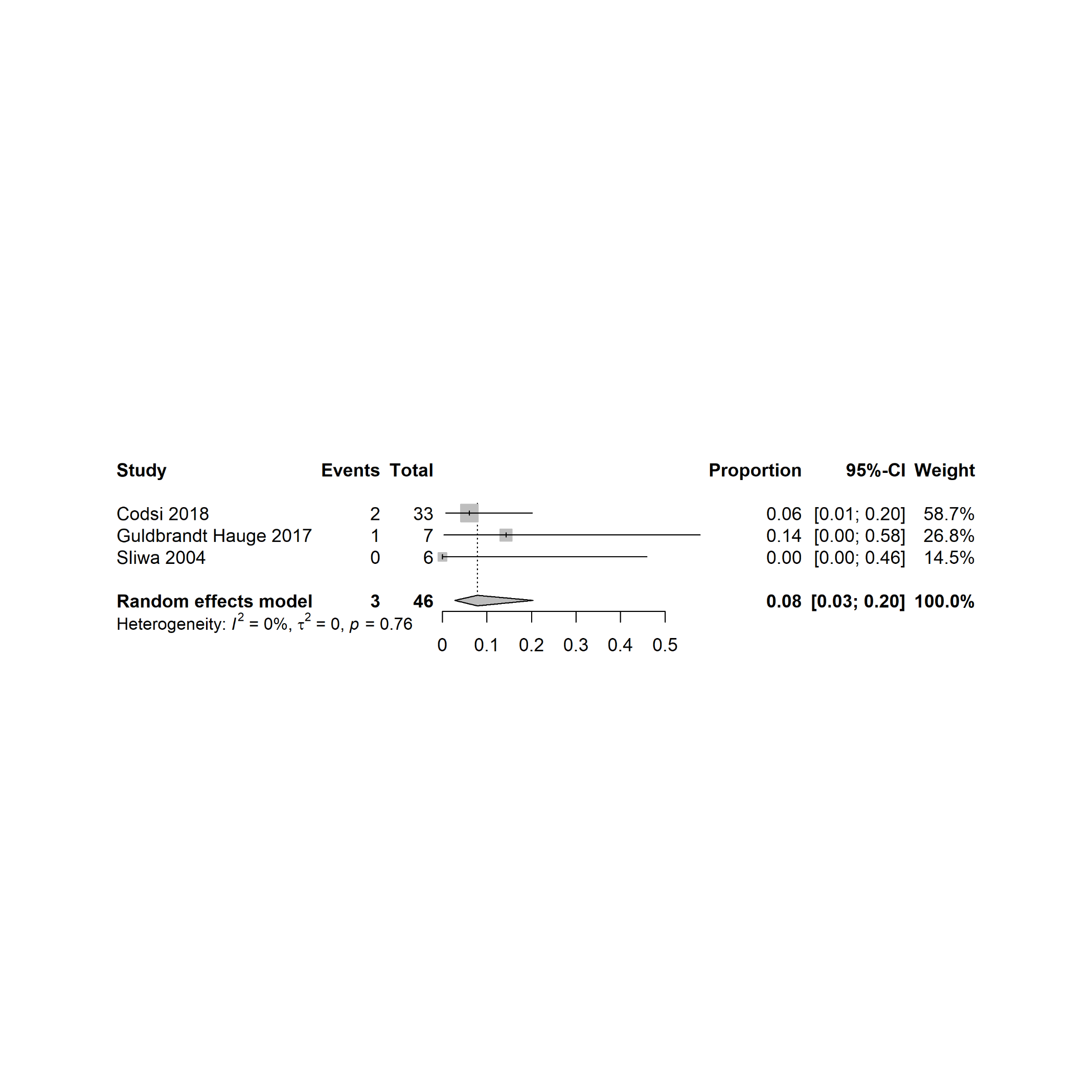


Figure S3e: Forest plot to show to the proportion of pregnancies resulting in pregnancy induced hypertension in subsequent pregnancies after peripartum cardiomyopathy


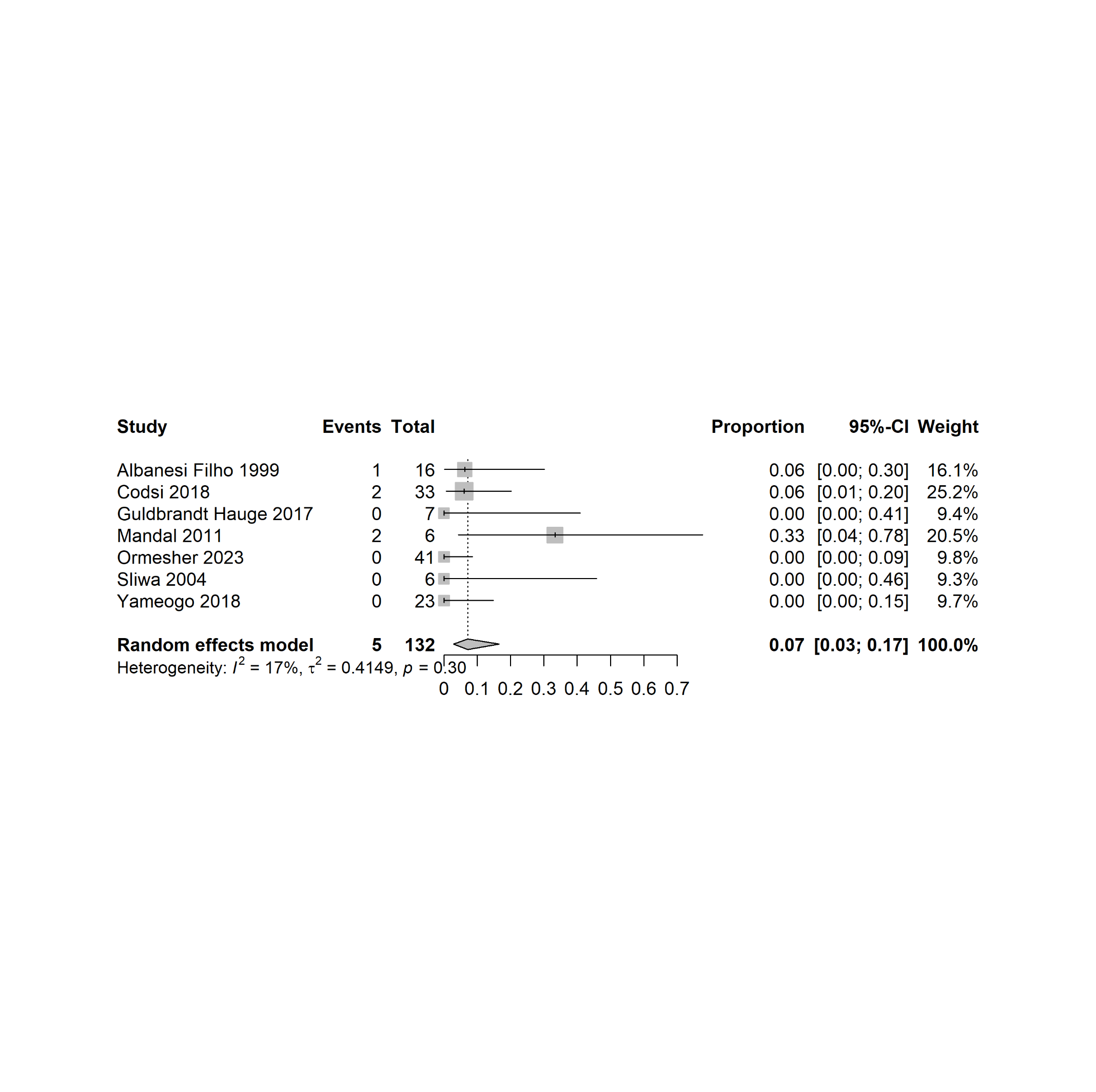


Figure S3f: Forest plot to show to the proportion of pregnancies resulting in pre-eclampsia in subsequent pregnancies after peripartum cardiomyopathy


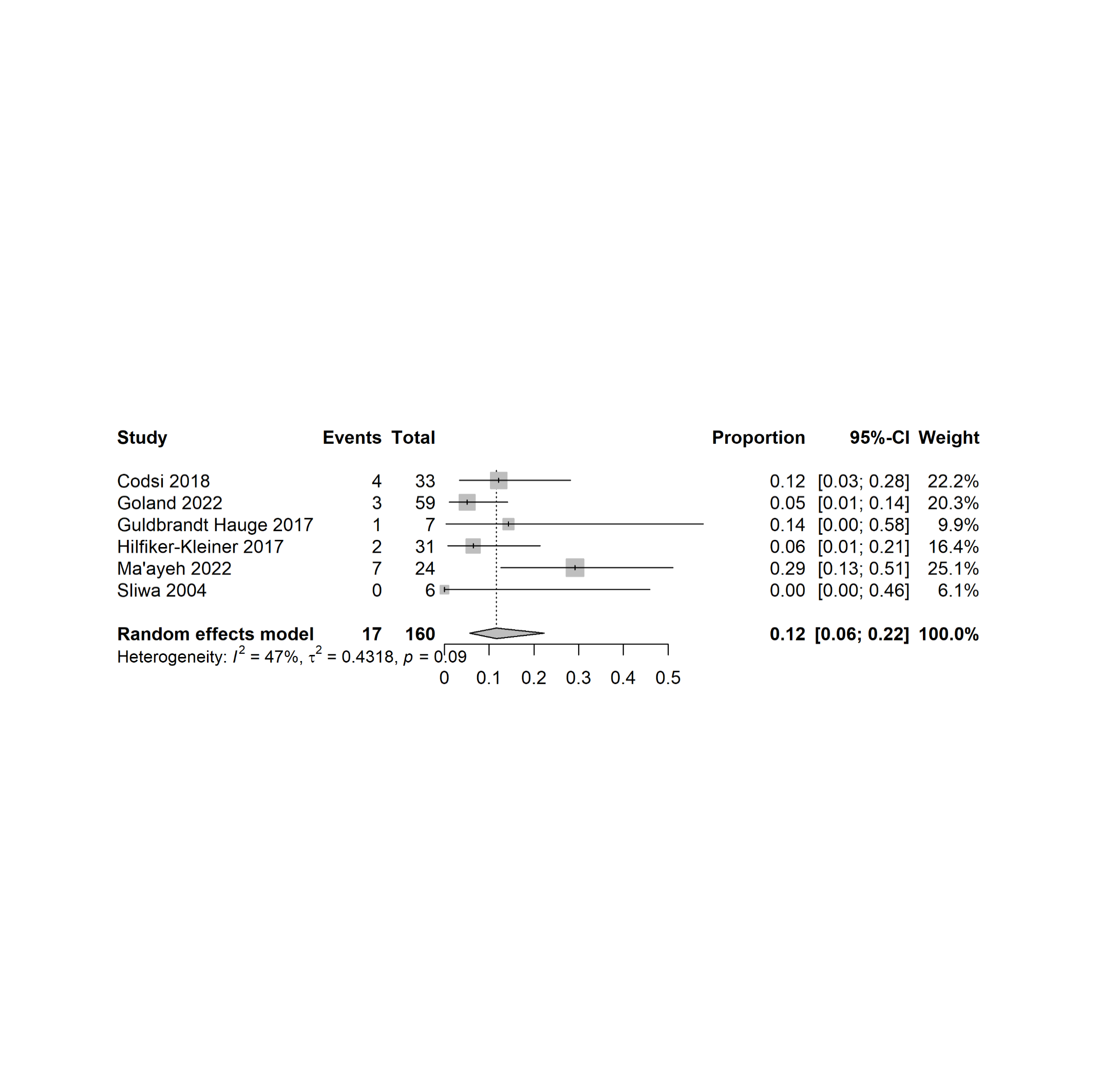


Figure S3g: Forest plot to show to the proportion of pregnancies resulting in hypertensive disorders (composite of pre-eclampsia and pregnancy induced hypertension) in subsequent pregnancies after peripartum cardiomyopathy


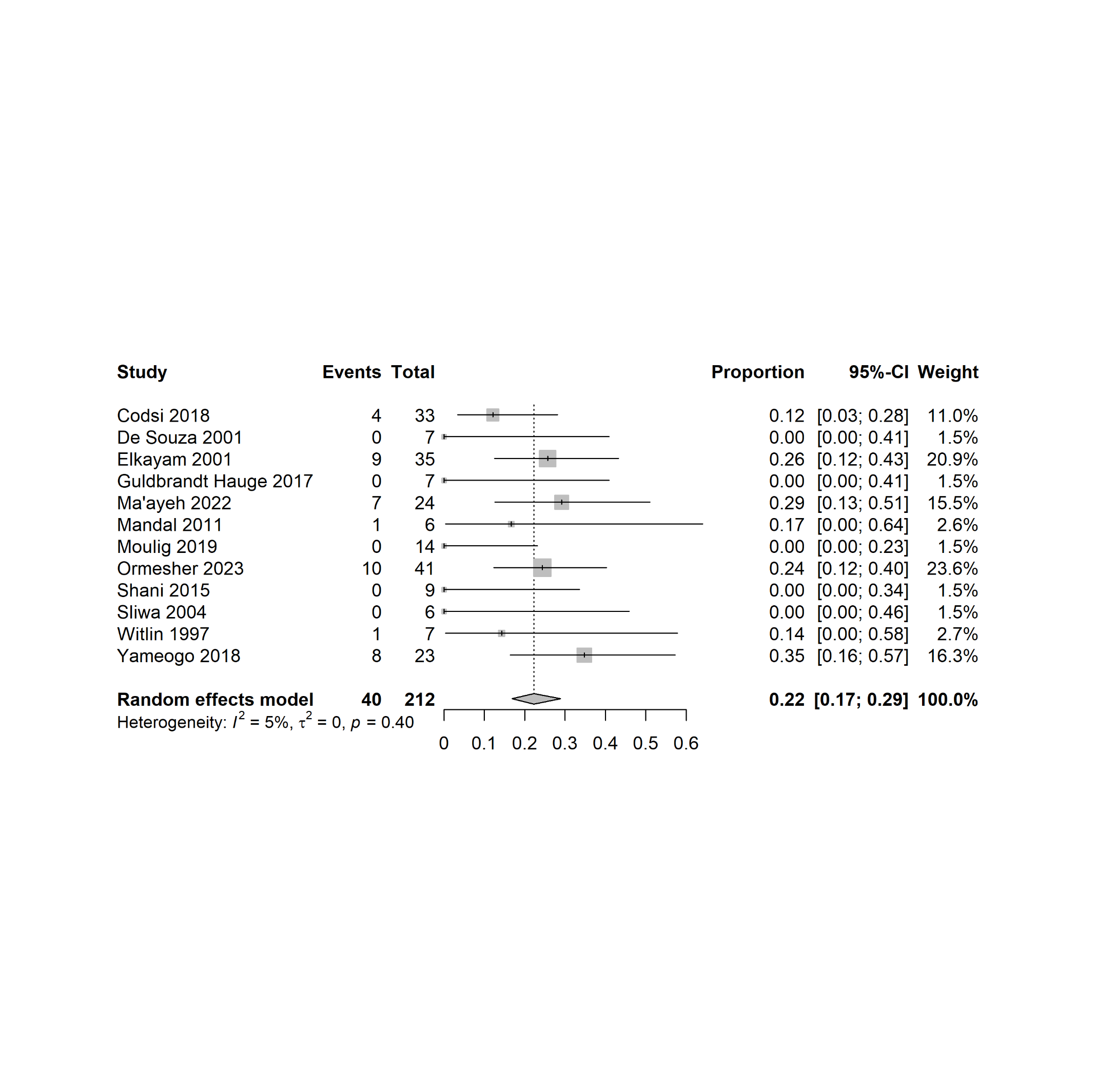


Figure S3h: Forest plot to show risk of preterm birth <37 weeks in subsequent pregnancies after peripartum cardiomyopathy


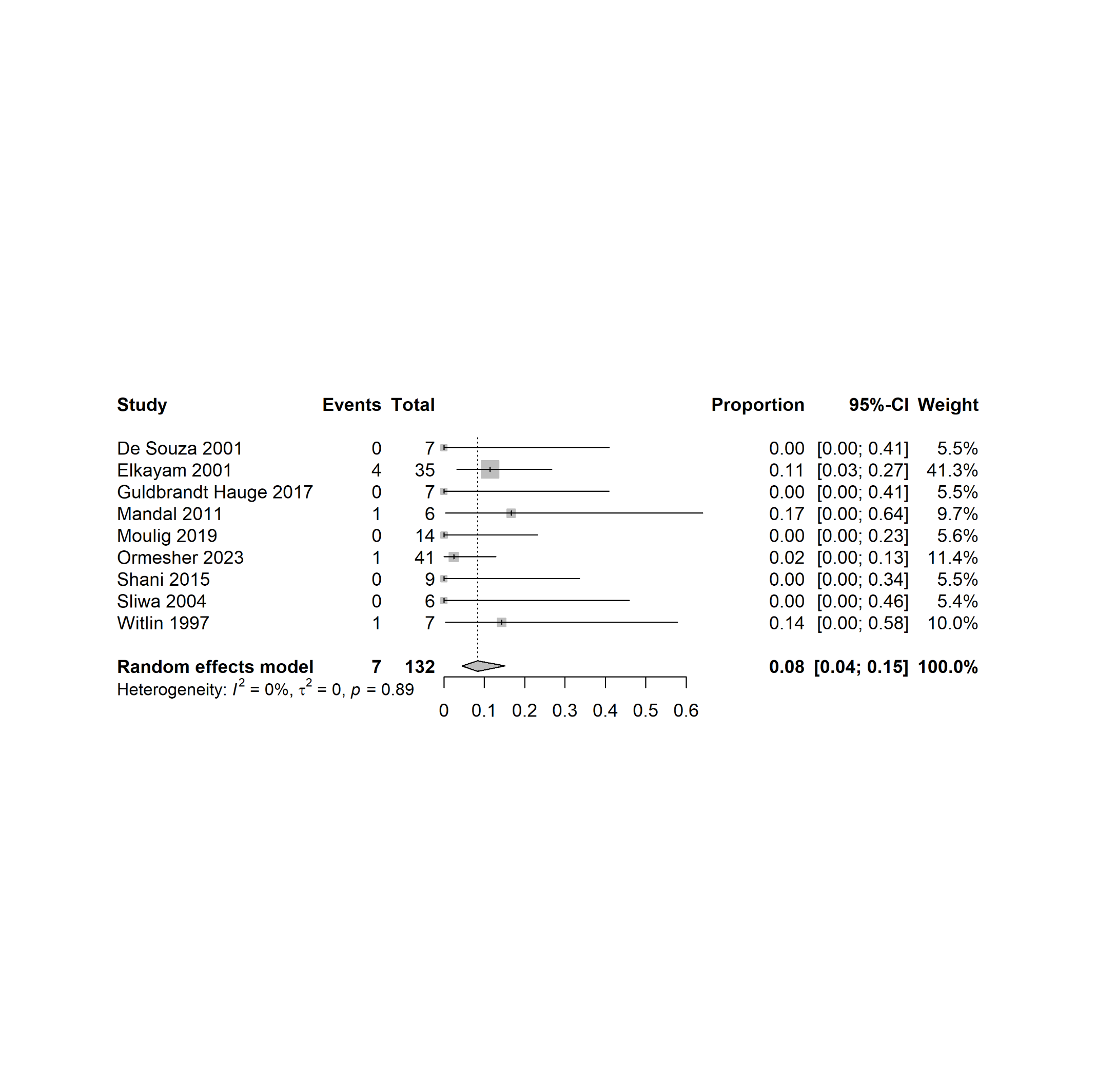


Figure S3i: Forest plot to show risk of preterm birth <34 weeks in subsequent pregnancies after peripartum cardiomyopathy


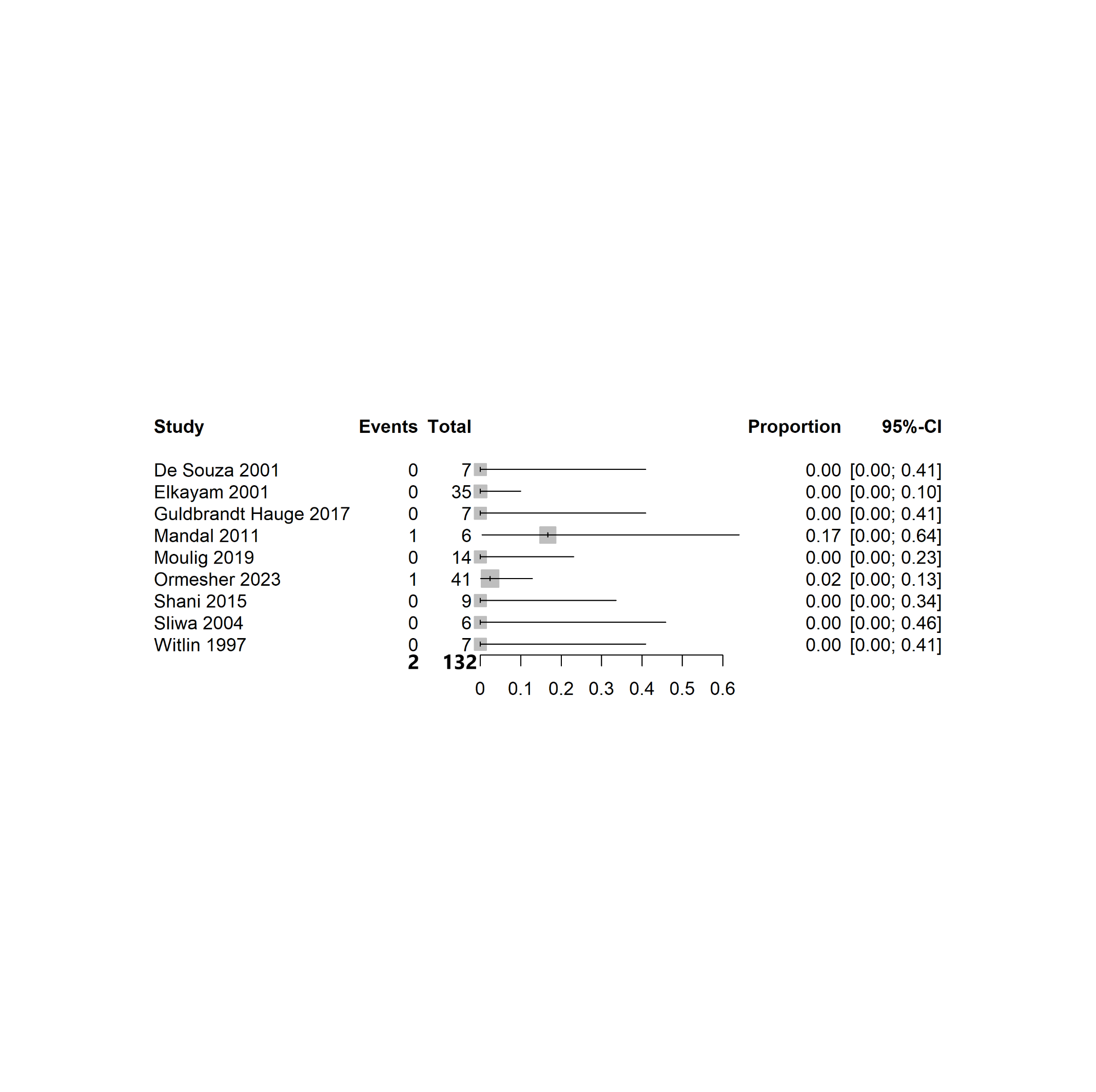


Figure S3j: Forest plot to show risk of preterm birth <28 weeks in subsequent pregnancies after peripartum cardiomyopathy


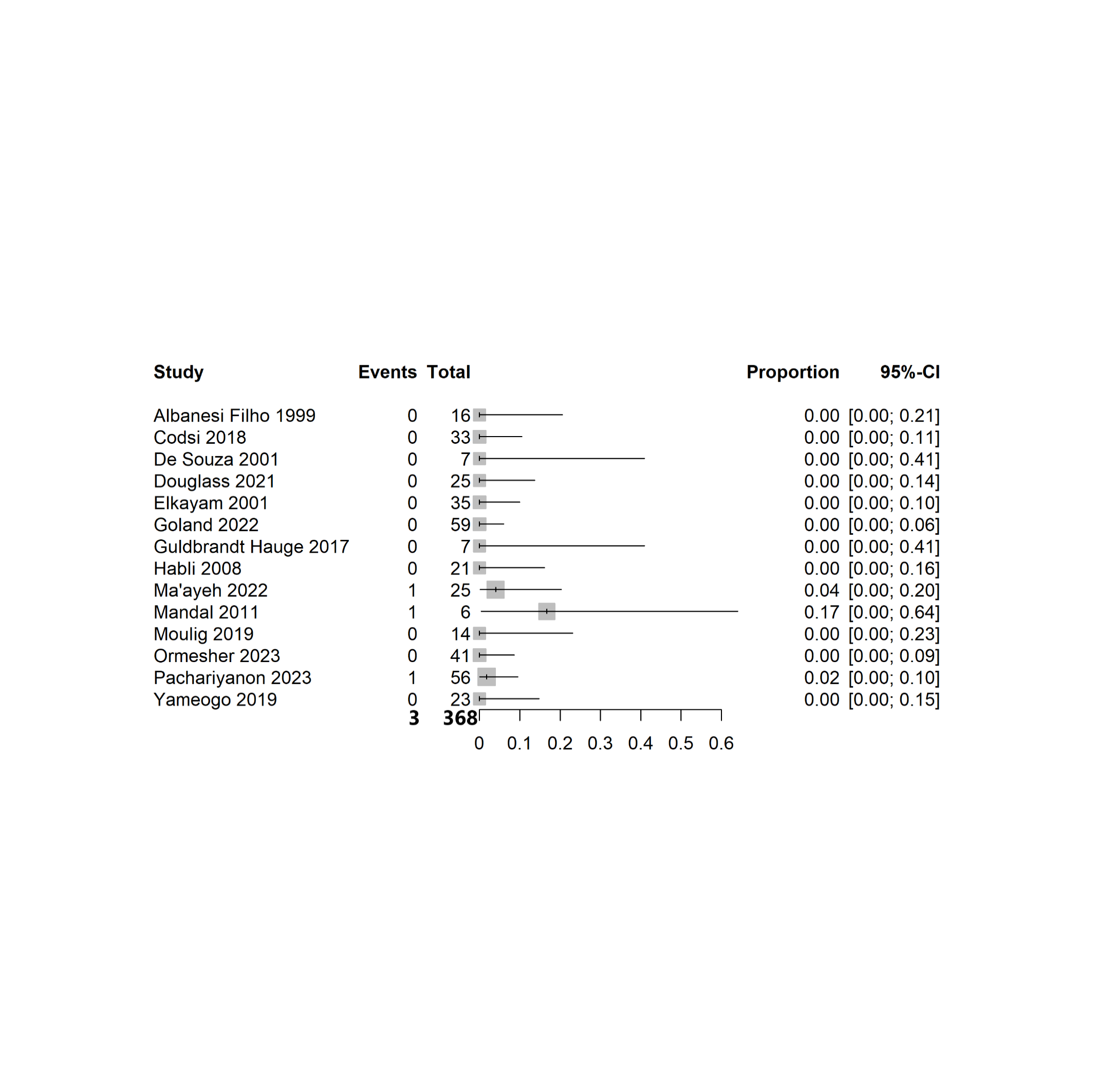


Figure S3k: Forest plot to show risk of stillbirth in subsequent pregnancies after peripartum cardiomyopathy


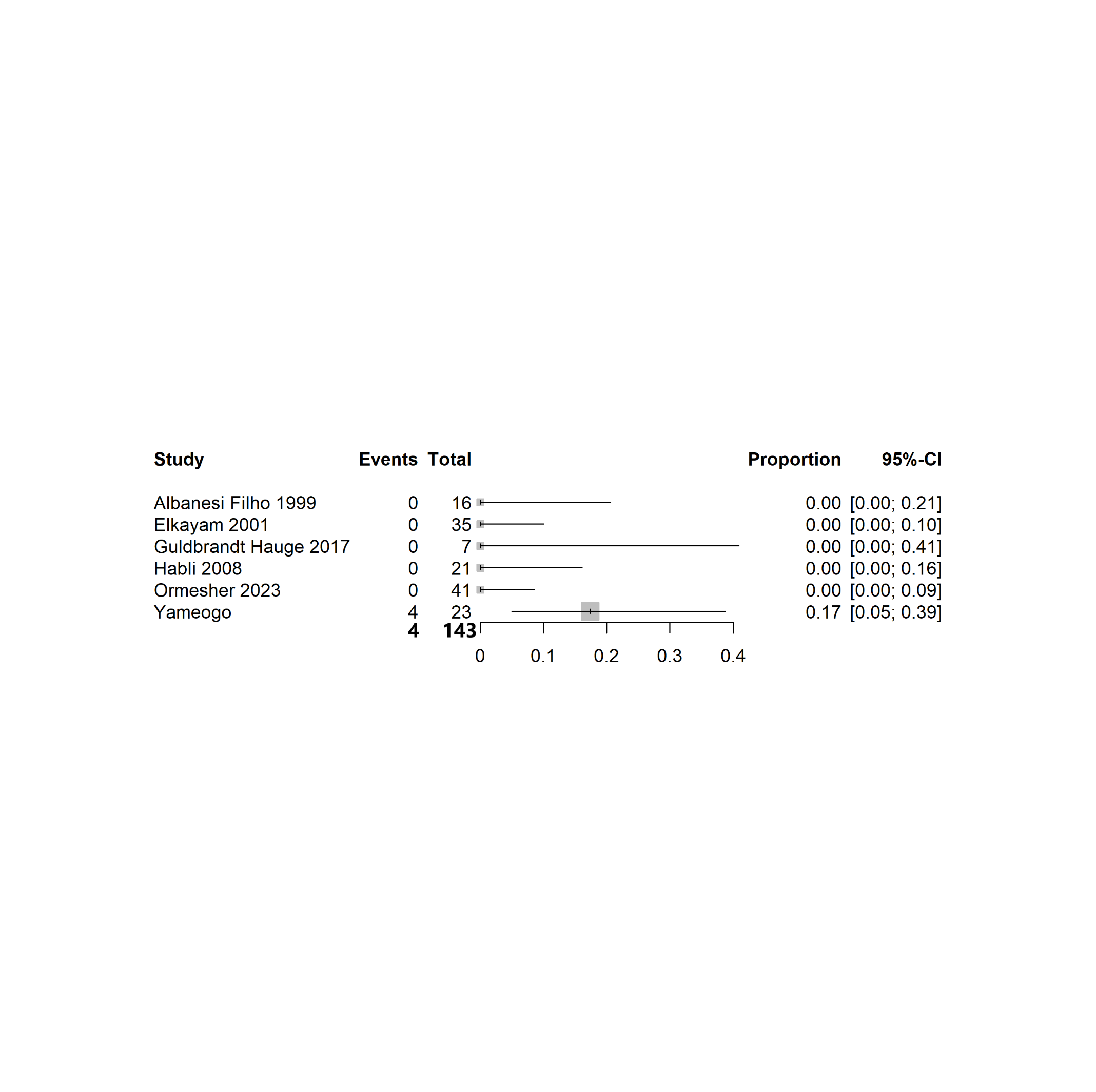


Figure S3l: Forest plot to show risk of neonatal death in subsequent pregnancies after peripartum cardiomyopathy


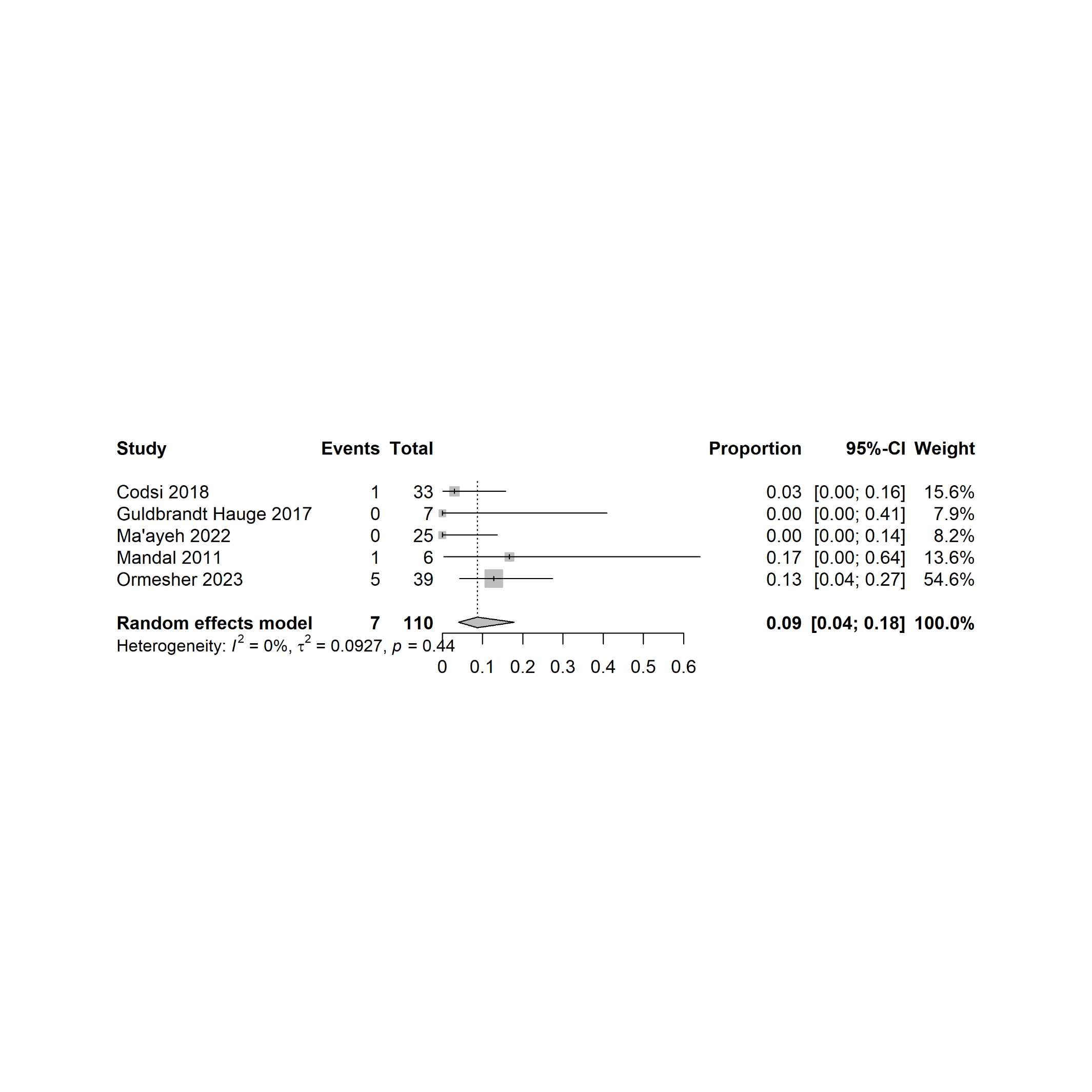


Figure S3m: Forest plot to show risk of fetal growth restriction in subsequent pregnancies after peripartum cardiomyopathy


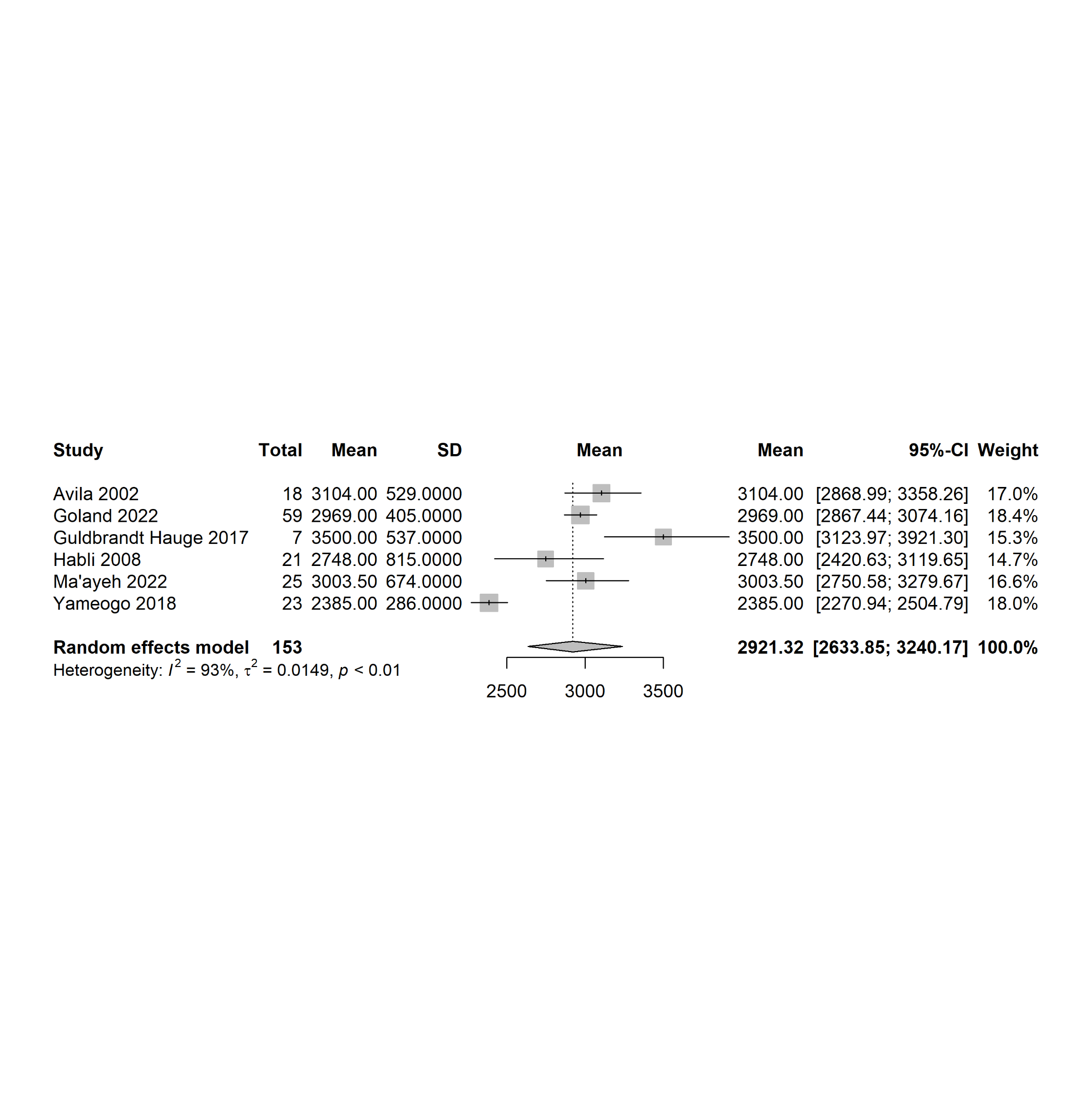


Figure S3n: Forest plot to show mean birthweight (grams) in subsequent pregnancies after peripartum cardiomyopathy


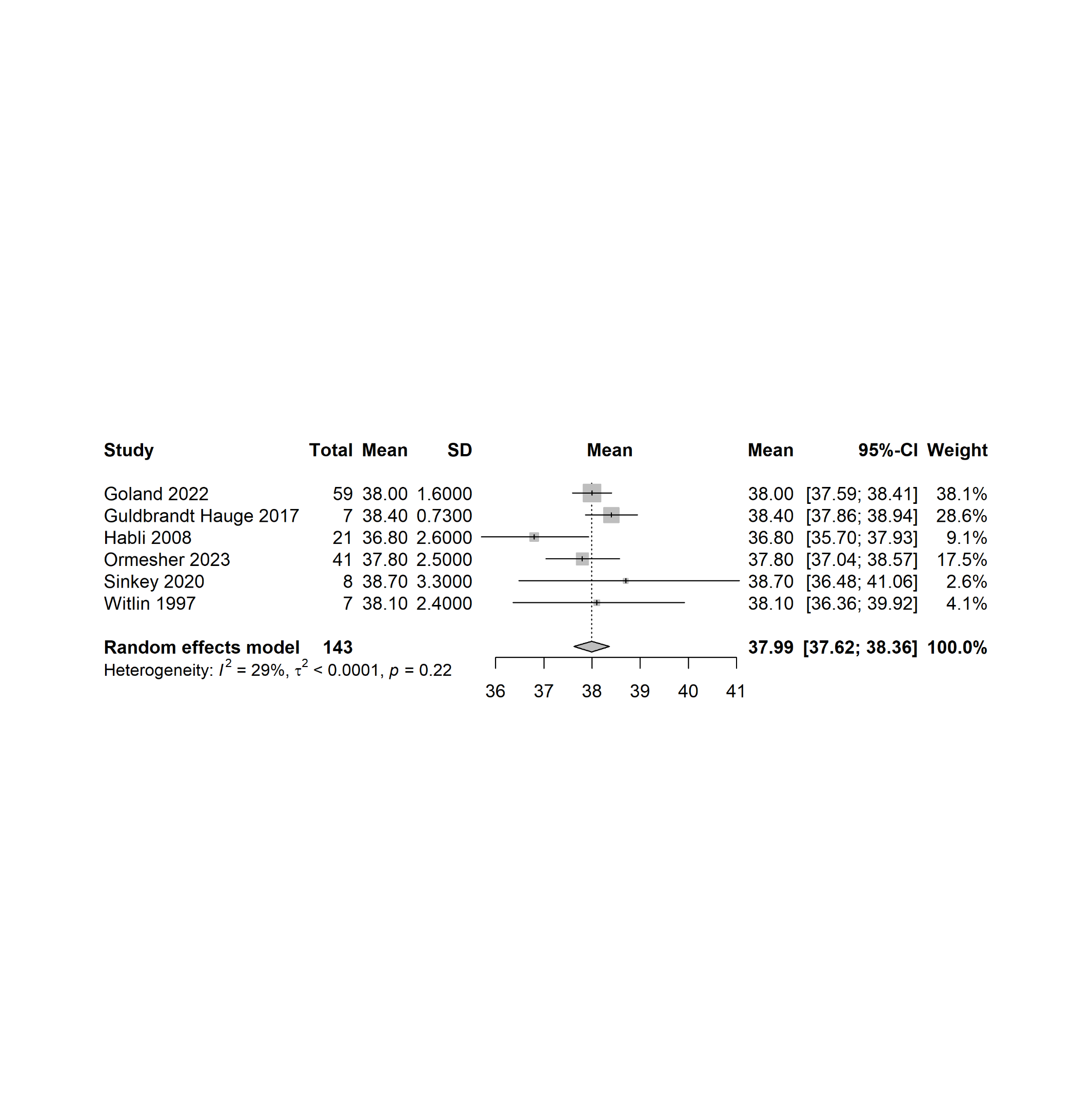


Figure S3o: Forest plot to show mean gestational age at birth (weeks) in subsequent pregnancies after peripartum cardiomyopathy
